# ALS is imprinted in the chromatin accessibility of blood cells

**DOI:** 10.1007/s00018-023-04769-w

**Published:** 2023-04-24

**Authors:** Julia K. Kühlwein, Wolfgang P. Ruf, Katharina Kandler, Simon Witzel, Christina Lang, Medhanie A. Mulaw, Arif B. Ekici, Jochen H. Weishaupt, Albert C. Ludolph, Veselin Grozdanov, Karin M. Danzer

**Affiliations:** 1 Department of Neurology, University Clinic, University of Ulm, Albert-Einstein-Allee 11, 89081 Ulm, Baden-Wuerttemberg Germany; 2 Medical Faculty, University of Ulm, 89081 Ulm, Baden-Wuerttemberg Germany; 3 Institute of Human Genetics, University Clinic Erlangen, Friedrich-Alexander-University Erlangen-Nürnberg, 91054 Erlangen, Bayern Germany; 4 Division for Neurodegenerative Diseases, Neurology Department, University Medicine Mannheim, Heidelberg University, 68167 Mannheim, Baden-Wuerttemberg Germany; 5 German Center for Neurodegenerative Diseases (DZNE), 89081 Ulm, Baden-Wuerttemberg Germany

**Keywords:** Chromatin remodeling, Epigenome, Integrated analysis, Motor neuron disease, Regulatory elements, Single-nuclei sequencing

## Abstract

Amyotrophic Lateral Sclerosis (ALS) is a complex and incurable neurodegenerative disorder in which genetic and epigenetic factors contribute to the pathogenesis of all forms of ALS. The interplay of genetic predisposition and environmental footprints generates epigenetic signatures in the cells of affected tissues, which then alter transcriptional programs. Epigenetic modifications that arise from genetic predisposition and systemic environmental footprints should in theory be detectable not only in affected CNS tissue but also in the periphery. Here, we identify an ALS-associated epigenetic signature (‘*epiChromALS*’) by chromatin accessibility analysis of blood cells of ALS patients. In contrast to the blood transcriptome signature, epiChromALS includes also genes that are not expressed in blood cells; it is enriched in CNS neuronal pathways and it is present in the ALS motor cortex. By combining simultaneous ATAC-seq and RNA-seq with single-cell sequencing in PBMCs and motor cortex from ALS patients, we demonstrate that epigenetic changes associated with the neurodegenerative disease can be found in the periphery, thus strongly suggesting a mechanistic link between the epigenetic regulation and disease pathogenesis.

**Supplementary Information:**

The online version contains supplementary material available at 10.1007/s00018-023-04769-w.

## Introduction

Sampling of affected tissues in an easy-obtainable manner is crucial for understanding and treating diseases. Although the investigation of post-mortem tissues can be very informative, it is not appropriate for the study of prodromal disease mechanisms, early disease stages, or for longitudinal observation of the disease course. Hence, sample collection from living patients is essential. In Amyotrophic Lateral Sclerosis (ALS), sampling of the primarily affected tissue, the central nervous system (CNS), is not feasible in the living patient. Thus, easy-attainable sampling in the periphery that recapitulates pathological changes of the CNS is highly needed. Several studies have demonstrated functional and transcriptomic aberrations in ALS in peripheral blood mononuclear cells (PBMCs), which are easily obtained [1–5]. However, the altered function or transcriptional dysregulation of ALS PMBCs reflect processes specific to these cells, like immune functions, and are heavily influenced by variations in cell-type composition in ALS; lastly, they are limited to genes expressed in blood. Here, we hypothesized that some of these limitations may be overcome by studying the epigenome of PBMCs in ALS by assaying the chromatin accessibility. Chromatin accessibility is the cumulative product of complex genetic predisposition and epigenomic alterations (epigenome). The epigenome can be considered as a dynamic template that adapts flexibly to diverse external stimuli without altering the genetic code and that shapes gene expression. Over the lifetime, different environmental stimuli will be imprinted in the epigenome and render it more prone to pathogenic alterations [6–9]. In line with this, aging is one of the most important risk factors for ALS and acceleration of epigenetic aging in ALS, measured by DNA methylation in blood, has been linked to an earlier age of onset and faster progression of ALS [10–12]. Thus, we speculated that if genetic predisposition and systemic environmental stimuli shape the epigenomic landscape in ALS, an ALS-specific epigenetic signature should be detectable not only in CNS but also in PBMCs. To test this hypothesis, we investigated the genome-wide chromatin accessibility of PBMCs in patients with sporadic ALS (sALS) by assay for transposase-accessible chromatin using sequencing (ATAC-seq) in bulk and at the single-cell level. We then assayed and compared the PBMC transcriptome and the chromatin accessibility of the ALS brain and integrated the findings with multi-omic ALS data.

## Methods

### Study cohorts and ethical approval

All human experiments were performed in accordance with the declaration of Helsinki and approved by the Ethics Committee of Ulm University [13]. Informed consent was obtained from all participants included in the study. ALS patients were diagnosed according to the El-Escorial revised criteria for ALS [14] and recruited at the University Clinic of Ulm. Patients without any indication of a familial history were considered as sporadic. In addition, a specific genotyping for patients of the bulk ATAC and RNA study was conducted for 12 out of 23 ALS patients with a panel screening for ALS-associated mutations (*ALS2, ANG, ARHGEF28, ATXN2, BSCL2, CCNF, CHCHD10, CHMP2B, DCTN1, ERBB4, FIG4, FUS, GBE1, GLE1, GRN, HNRNPA1, HNRNPA2B1, HSPB1, HSPB8, MAPT, MATR3, MME, NEFH, NEK1, OPTN, PFN1, PRPH, SETX, SIGMAR1, SOD1, SPG11, SPG20, SQSTM1, TAF15, TARDBP, TBK1, TUBA4A, UBQLN2, VAPB, VCP, VEGFA, VPS54)* as well as for the *C9orf72 HRE* by repeat primer PCRs. 11 ALS patients could not be tested either because they did not enroll for genetic testing or they passed away before testing was conducted. Healthy controls (HCs) without neurological conditions were selected to match the ALS cohort in age and  sex. A summary of all clinical and demographic characteristics of the participants is provided in Tables S1 & S2. From all participants, whole venous blood was collected in a standard Monovette blood drawing system (Sarstedt) containing EDTA as an anticoagulant. Blood samples were processed within one hour after blood collection.

Human post-mortem motor cortex samples from 3 ALS patients were fresh, flash-frozen without any fixation. A detailed genotyping of brain tissue was done for 42 ALS-causative genes in a panel as described above and for *C9orf72 HRE* by repeat primer PCR following Southern Blot confirmation.

### PBMC isolation

PBMCs from whole blood were isolated using Histopaque™-1077 density gradient centrifugation. Typical yields were ~ 1 × 10^6^ PBMCs per ml of blood. After washing the cells twice with DPBS, the PBMCs were used for ATAC-seq and RNA-seq.

### ATAC-seq

ATAC-seq protocol has been adapted from Buenrostro et al. [15]. Freshly isolated PBMCs (1 × 10^5^ cells) were pelleted at 500×*g* for 20 min at 4 °C. Immediately after lysis of the cells in 100 µl lysis buffer (10 mM Tris–HCl [pH 7.4], 10 mM NaCl, 3 mM MgCl_2_, 0.1% NP-40), the cells were centrifuged at 500 × *g* for 20 min and 4 °C. Pellets were resuspended in 50 µl transposition buffer (25 µl 2 × Tagment DNA buffer (Illumina), 2.5 µl Tn5 enzyme (Illumina), 22.5 µl nuclease-free H_2_O) and incubated for 30 min at 37 °C. Tagmented DNA was then purified using Qiagen MinElute Reaction Cleanup kit (QIAGEN) and amplified using NEBNext High-Fidelity 2 × PCR master Mix (NEB), barcoded primers (Metabion) and the following cycling conditions: 72 °C for 5 min, 98 °C for 30 s, followed by 5 cycles of 98 °C for 10 s, 63 °C for 30 s and 72 °C for 1 min. Amplification optimization was performed by qRT-PCR with a small aliquot of the reaction mix. In total, PCR reactions were terminated after 6–12 cycles. Next, libraries were purified and size-selected using AMPure XP beads (Beckman Coulter). Libraries were assessed on a Tape Station2200 (Agilent) and BioAnalyzer (Agilent) for evaluating the size distribution and by qRT-PCR-based quantification (KAPA library quantification kit, Roche). Libraries were sequenced on a NovaSeq 6000 S4 flow cell (Illumina) with a minimum of 60 million raw paired-end reads with a length of 150 bp per library.

### ATAC-seq data analysis

The quality of raw sequencing data was assessed with FastQC (v.0.11.9) [16]. ATAC-seq reads were aligned, filtered, peaks called and library qc assessed with the ENCODE ATAC-seq pipeline (v2.1.3) [6]. Briefly, adapter sequences were trimmed with cutadapt v3.2 *[-m 5 -e 0.2]* [17], reads aligned to the human genome *(GRCh38.p13 no_alt assembly)* with bowtie2 v2.2.6 *[-X 2000, -k 0]* [18], quality assessed with MultiQC [19], ataqv (v1.2.1) [20] and deeptools (v2.0) [21], PCR- and optical duplicates, low-quality reads and mitochondrial reads filtered out with samtools (v.1.2) [22], sambamba (v0.6.5) [23] and Picard tools (v 1*.*26) [24]. TagAlign files were generated with gawk by treating paired-end as single-end reads and shifting reads 5 bp (+ strand) and 4 bp (- strand). Regions of high chromatin accessibility *(‘peaks’)* were called from the TagAlign files with MACS2 v2.1.0 *(effective genome size* = *2.70e* + *09, band width* = *300, –shift -75, –extsize 150 –nomodel –SPMR –call-summits -p 0.01)* [25] and filtered for blacklisted regions *(ENCODE)*. Consensus peaks were then generated with BEDtools by merging overlapping peaks from all different samples and keeping all peaks that were called separately in at least two samples (bedtools multiinter, and bedtools merge v.2.22) [26]. Counts in consensus peaks were counted with featureCounts (v1.6.0) [27]. Peaks were annotated by proximity with HOMER (v4.11) [28], ChIPseeker (v1.32) [29] and by association with expression with Signac (v1.5.0) [30], function “LinkPeaks” with default parameters (distance for genes to consider: 500 Kbp up/down-stream from a peak) in two different multiomic datasets: one that was composed of the bulk ATAC-seq data and the bulk RNA-seq data from this study, where each observation point for the correlation is an individual (HC/ALS patient); and the 10X Genomics sorted PBMCs Multiome ATAC + GEX dataset (10X Genomics) from one individual, where every observation point for the correlation is a cell/metacell. All bulk ATAC-seq samples satisfied the primary QC metrics for bulk ATAC-seq data by ENCODE of transcription start site (TSS) enrichment > 8 (for GRCh38) or fraction of reads in peaks (FRiP). Genomic regions (peaks) were compared and intersected with GenomicRanges (v1.48.0) [31]. Differential peak accessibility was calculated with DESeq2 (v1.32.0) with Wald’s test [32]. Weighted Gene Correlation Network Analysis (WGCNA) was performed with WGCNA (v1.71) [33] with DESeq2-normalized counts. Since only the 729 differentially accessible regions between healthy controls and ALS patient were analyzed, only ALS patients, but not healthy controls were included in the analysis to avoid violating the assumption of independence of the features from the sample groups. The weighted module accessibility per sample was calculated by weighting the accessibility of each peak by its significance for the respective module.

### RNA-seq

Total RNA was isolated from PBMCs of ALS patients and HCs using the Rneasy Plus Mini Kit (Qiagen) according to the manufacturer’s instructions including an additional Dnase I digestion. The quantity of RNA was assessed by spectrometry using the NanoDrop-2000 (Thermo Fisher Scientific) as well as the 2100 Bioanalyzer (Agilent). RIN (RNA Integrity Number) of all samples was ≥ 8.0. For RNA-seq, dual-indexed libraries were generated from 1 µg high-quality RNA using the Illumina TruSeq stranded mRNA kit (Illumina). Libraries were subjected to single-end sequencing (101 bp) on a HiSeq-2500 platform (Illumina). The obtained reads were demultiplexed and converted to FASTQ format using bcl2fastq (v2.17.1.14). Quality filtering and removal of adapter sequences were performed with cutadapt (v3.2). Reads shorter than 60 bp following adapter trimming were removed. Read quality was determined before and after adapter trimming with FastQC (v0.11.8).

### RNA-seq data analysis

Reads were aligned to the human genome (GRCh38.p13) using TOPHAT2 (v2.1.1) [34] and the feature count matrix was generated with featureCounts (v1.6.0) [27], both with default settings. Differential analysis was performed with DESeq2 (v1.32.0) [32].

### Isolation of nuclei for single-cell ATAC-seq

*Nuclei from PBMCs* PBMCs were isolated as described previously. 1 × 10^6^ PBMCs were centrifuged at 500 × *g* for 10 min at 4 °C. The pellet was resuspended in 200 µl ice-cold 1: 5 diluted lysis buffer (10 mM Tris–HCl [pH 7.4], 10 mM NaCl, 3 mM MgCl_2_, 0.1% Tween-20, 0.1% NP-40, 0.01% Digitionin, 1% BSA, 1 mM DTT, 1 U/µl RNase inhibitor) and incubated for 3 min on ice. Afterwards, 500 µl ice-cold wash buffer (10 mM Tris–HCl [pH 7.4], 10 mM NaCl, 3 mM MgCl_2_, 1% BSA, 0.1% Tween-20, 1 mM DTT, 1 U/µl RNase inhibitor) was added immediately to the lysed cells and centrifuged at 500 × *g* for 10 min at 4 °C. Without disrupting the pellet, the supernatant was removed and the nuclei pellet was washed twice with 250 µl ice-cold wash buffer at 500×*g* for 10 min at 4 °C. After the last washing step, the nuclei were resuspended in 1 × nuclei buffer (1 × nuclei buffer from 10X Genomics (PN-2000207), 1 mM DTT, 1 U/µl RNase inhibitor). As a quality control, the nuclei number and morphology were determined upon DAPI staining and processed according to the Chromium Next GEM Single Cell ATAC user guide (CG000209 RevD; 10X Genomics).

Nuclei from brain tissue:

Nuclei were isolated from fresh, flash-frozen human brain tissue (post-mortem) as described before [35]. The final nuclei stock was resuspended in diluted nuclei buffer (1X nuclei buffer from 10X Genomics (PN-2000207), 1 mM DTT, 1 U/µl RNase inhibitor). As a quality control, the nuclei number and morphology were determined upon DAPI staining. Immediately after isolation, the nuclei were processed according to the Chromium Next GEM Single Cell ATAC user guide (CG000209 RevD; 10X Genomics) and the Chromium Next GEM Single cell Multiome user guide (CG000338 RevE; 10X Genomics).

### Generation of single-cell libraries and sequencing

Single-cell nuclei suspension was loaded onto the Chromium Controller using Chromium Next GEM Single Cell ATAC reagent kits v1.1 (10X Genomics). Sample processing and library preparation was performed according to the manufacturer’s instructions (CG000209 RevD from 10X Genomics), targeting 10,000 nuclei recovery per sample. Single-cell ATAC libraries were sequenced on a Novaseq 6000 SP flow cell with 50 bp paired-end reads. Healthy control single-cell ATAC-seq PBMC data was accessed from 10X Genomics under a CC-BY license (https://s3-us-west-2.amazonaws.com/10x.files/samples/cell-atac/1.2.0/atac_v1_pbmc_10k/atac_v1_pbmc_10k_fastqs.tar, https://s3-us-west-2.amazonaws.com/10x.files/samples/cell-atac/1.2.0/atac_v1_pbmc_5k/atac_v1_pbmc_5k_fastqs.tar, https://s3-us-west-2.amazonaws.com/10x.files/samples/cell-atac/1.2.0/atac_v1_pbmc_500/atac_v1_pbmc_500_fastqs.tar, https://s3-us-west-2.amazonaws.com/10x.files/samples/cell-atac/1.2.0/atac_v1_pbmc_1k/atac_v1_pbmc_1k_fastqs.tar).

### Single-cell ATAC-seq data analysis

Raw single-cell sequencing reads were processed with 10X Genomics Cell Ranger ATAC (v2.1). Single-cell sequencing data were analyzed with Seurat (v4.0.6) [36, 37], Signac (v1.5.0) [30] and Scanpy (v1.9.1) [38]. Barcodes that were identified by Cell Ranger ATAC as cells were retained and filtered further for cells with un-proportionally high or low QC parameters (FRiP < 0.15, peak region fragments > 20,000 or < 1000, TSS < 2 and nucleosome signal > 4). Different samples were merged by generating consensus peaks, generating feature barcode matrices and integration with Signac *[findIntegrationAnchors (2–30 rlsi components, 55 k.anchors) and IntegrateEmbeddings]*. Dimensionality reduction and clustering were performed with Signac and Seurat *[RunTFIDF, FindTopFeatures(top 90%), RunSVD, RunUMAP, FindNeighbors and FindClusters]* with up to 30 principal components, excluding principle components highly associated with read depth. Gene activity was predicted from peak data with Signac *[GeneActivity]* and feature block expression/accessibility was calculated with Seurat *[AddModuleScore, 50–100 controls features]* or Signac *[AddChromatinModule]*. Cell-type identities were annotated based on the predicted gene activity of canonical cell markers. The chromatin accessibility of the 764 683 peaks detected in bulk ATAC-seq in scPBMCs was assessed by generating a cell-feature-count matrix for these peaks with Seurat and Signac *[FeatureMatrix, CreateChromatinAssay]*. Differential accessibility in a cell-wise manner for the analysis of the dependence on cell types was performed with Seurat *[FindMarkers with Logistic Regression and peak region fragments as latent variables]*. Differential accessibility of the 729 bulk ATAC-seq peaks that were differentially accessible in ALS patients’ PBMCs was analyzed in a targeted approach while controlling for the effective library sizes considering all 764 683 peaks with Seurat with Libra (v1.0.0) for pseudo-bulk comparisons *[run_de with de_family* = *”pseudobulk”, de_method* = *”DESeq2″, de_type* = *”LRT”]* with an FDR cutoff of q < 0.05 for considering a difference statistically significant [39]. Overlap of genomic regions between bulk and single-cell data was calculated with GenomicRanges (v1.48.0) [31].

### Single-cell Multiome ATAC-seq + GEX data analysis

Single-cell Multiome ATAC-seq + GEX data was accessed from 10X Genomics under a CC-BY license (Human 1 × HC PBMCs Multiome ATAC + GEX dataset, PBMCs sorted to remove granulocytes, https://www.10xgenomics.com/resources/datasets/pbmc-from-a-healthy-donor-granulocytes-removed-through-cell-sorting-10-k-1-standard-2-0-0) and processed with Seurat (v4.0.6) [36, 37] and Signac (v1.5.0) [30]. A cell-feature-count matrix for the 764 683 peaks detected in bulk ATAC-seq was generated with Seurat and Signac *[FeatureMatrix, CreateChromatinAssay]*. Quality of the cell-feature matrix was controlled by comparing it to the feature matrix for the peaks called by CellRanger on this dataset. Cells were filtered for quality control with following parameters: *nCount_ATAC* > *1e4 & nCount_ATAC* < *1e5 & nCount_RNA* > *1e3 & nCount_RNA* < *2.5e4 & nucleosome_signal* < *2 & TSS.enrichment* > *1*. ATAC-seq data was normalized with TF-IDF *[RunTFIDF]* [40]. Cell-type identities were annotated by integration of the gene expression data with a publicly available dataset of > 150 000 PBMCs, which were precisely annotated by single-cell gene expression and surface antigen markers (GSE164378, GSE100866) [36, 41]. Gene expression data was normalized with SCT *[SCTransform]* [42]. Annotation of bulk-ATAC-seq peaks with genes by correlation of chromatin accessibility and gene expression was performed with Signac *[LinkPeaks]* with default parameters with the SCT-transformed gene expression data.

### Functional analysis and data visualization

Gene enrichment analysis was performed with limma (v3.48.3) [43], GO.db (v3.13.0) [44] and Panther (v16.0). Further functional annotations were accessed from the GO database (release 12/2021) [45, 46], the Harmonizome database [47], GWASdb database [48], biomaRt (v2.48.3) [49], AnotationDbi (v1.54.1) [50] and the String and PEREGRINE databases. Data were visualized with ggplot2 (v3.3.5) [51], tidyverse (v1.3.2) [52], patchwork (v1.1.2), pheatmap (v1.0.12) [53], Seurat (v4.0.6) [36, 37] and viridis (v0.6.2). Venn diagrams were plotted with the Venn diagram tool, University of Gent (http://bioinformatics.psb.ugent.be/webtools/Venn). Genomic tracks were visualized with the IGV genome browser [54]. Cell-type abundance was estimated with ABIS [55]. Tissue specificity of genes of interest was assessed with data from the Human Protein Atlas accessed over the HPA API under R [56]. Random sets of ATAC peaks and genes were generated with R 4.1.1 (R Core Team) with seed *‘3,141,592’*. The random permutation of study volunteers was designed iteratively while monitoring multiple QC and cohort characteristics to balance the two random control groups for age, sex, disease condition, sequencing metrics and ATAC-seq QC data.

### Statistical analysis

Statistical analysis was performed with R 4.1.1 (R Core Team). Normal distribution was tested with D’Agostino and Pearson omnibus normality tests, Shapiro–Wilk normality test and Kolmogorov–Smirnov normality test. Hypothesis testing was performed with Mann–Whitney *U*-test and Student’s *t*-test for group-wise comparisons and with Wald’s test for differential accessibility/expression. The false discovery rate in multiple testing was controlled with the Benjamini–Hochberg FDR. Correlation was tested with Spearman’s and Pearson’s correlation tests. Enrichment analyses were tested with Fisher’s exact test with FDR correction. Differences in proportions in contingency tables were tested with the 2-sample test for equality of proportions with continuity correction.

## Results

### ATAC-seq identifies an ALS-associated chromatin accessibility signature in PBMCs

**Fig. 1:**
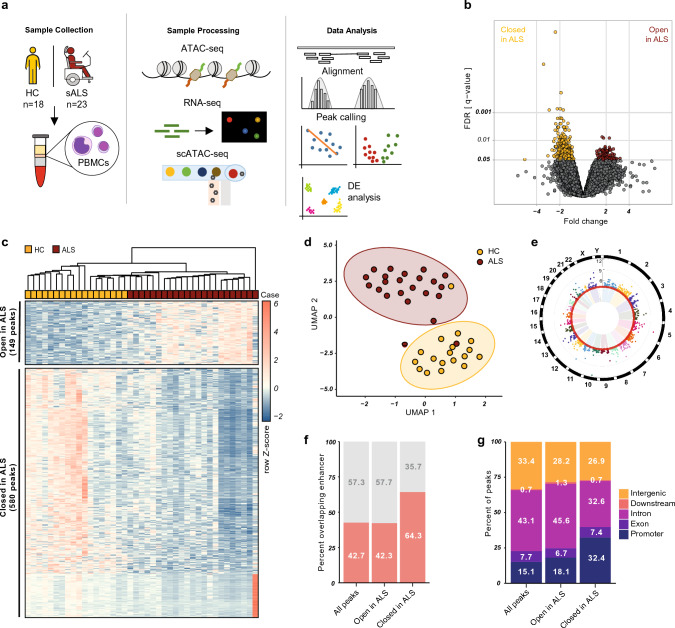
Chromatin accessibility signature of ALS PBMCs. **a** Schematic workflow of the study. PBMCs were isolated from HCs and ALS patients and subjected to bulk ATAC-seq and bulk RNA-seq in parallel or to single-cell ATAC-seq. **b** Differential chromatin accessibility analysis detected 729 genomic regions that are significantly (*q* < *0*.05, FDR) differentially accessible in ALS: 580 less accessible in ALS and 149 more accessible in ALS. **c** Hierarchical clustering of ALS patients and HCs according to the chromatin accessibility profile of the 729 differentially accessible peaks (WPGMA Average linkage with Euclidean distance). **d** UMAP projection of samples based on the differentially accessible peaks clearly separated ALS and HC samples. **e** Differentially accessible peaks are distributed over all autosomes and the X chromosome. **f**, **g** Differentially less accessible genomic regions in ALS are enriched in enhancers and in promoters (****p < *0.*0001, 2-sample test for equality of proportions with continuity correction)

To investigate disease-associated changes in chromatin accessibility and the transcriptome, we collected peripheral venous blood from 23 sALS patients *(f/m* = *8/15; mean age 65.8* ± *12.2 y)* and 18 age- and sex-matched HCs *(f/m* = *8/10; mean age 63.1* ± *9.4 y)*, isolated PBMCs and generated simultaneously bulk ATAC-seq and RNA-seq profiles from the same samples (Fig. 1a). Detailed information of the study cohort is summarized in Tables S1 (bulk ATAC & RNA-seq) and S2 (single-cell ATAC & RNA-seq).

Using ATAC-seq, we identified > 764,000 genomic regions with a significant chromatin accessibility signal *(‘peaks’)* across all 41 samples. All samples had a well-pronounced fragment size patterning that is typical for ATAC-seq libraries and had comparable ratios of nucleosome-free to mono-nucleosomal signal (Suppl. Figs. S1 & S2), as well as characteristic TSS enrichment profiles (Suppl. Fig. S3). The peaks represent genomic regions of open chromatin, which is accessible for the binding of gene regulatory elements and the transcriptional machinery. In bulk ATAC-seq, the peaks result from the sum of the accessibility of the chromatin at a given position in all cells of a sample. While a strong signal indicates that most of the cells have open chromatin at this genomic site, a weaker signal means that less cells have chromatin open at this site. The ATAC-seq peaks mapped to all autosomal and both sex chromosomes and covered ~ 13% of the genome *(mean peak length 522* ± *326 bp, median peak length 424 bp, IQR: 305–603 bp; detailed sequencing and peak statistics in Table S3 and Fig. S4)*. Annotation by proximity to the next TSS assigned the ATAC peaks to a total of 24,081 genes *(42% of the known genes; 30 peaks per gene on average, Table S3)*. Among these genes, protein-coding genes were strongly enriched (82% in the ATAC peaks vs 34% in the whole genome assembly, ****p < *0.0001)*, supporting the functional relevance of the open chromatin regions. Approximately one-third *(34%)* of all peaks fell into regions outside of the genes, while the rest of the peaks were localized at promoters and bodies of genes. The proportion of peaks localizing to promoters was ~ 15% and consistent with the literature [57]. When comparing HC samples to ALS, several QC parameters including the total number of filtered ‘clean’ reads, distinct fragments, peaks and peaks normalized per sequencing depth per individual were all comparable between ALS patients and HCs (Suppl. Fig. S5 and Table S4). Differential abundance analysis revealed 729 differentially accessible peaks in ALS at FDR < 0.05 *(*~ *0.1% of all peaks)* (Fig. 1b & Table S5). Interestingly, there were many more peaks that were significantly less accessible *(‘closed’)* in ALS *(n* = *580,* ~ *80%*) than peaks that were significantly more accessible *(‘open’)* in ALS *(n* = *149, 20%)*, even though the majority of all peaks with a difference between HC and ALS (significant + not significant) was open in ALS *(63% open and 37% closed)* (Fig. S6). Thus, differential accessibility of peaks in ALS was specifically concentrated in peaks that are less accessible in ALS (****p < *0.0001)*. A shuffled permutation of study participants was used as a negative control and showed that despite the huge number of measured peaks, no peaks were differentially accessible at the same FDR cutoff *(q* < *0.05)* when the compared groups are balanced for a disease state, age, sex and QC parameters (Suppl. Fig. S6). Moreover, 685 of the 729 differential peaks (94%) were re-detected with the same threshold after including sex as a covariate in the differential accessibility analysis. Age was not included as a covariate, as it is tightly associated with the age of onset of ALS and with ALS in general. Supervised hierarchical clustering with all 729 differentially accessible peaks showed moderate discrimination of ALS patients from HCs and suggests the presence of subgroups in both cohorts (Fig. 1c, d & Suppl. Fig. S7). Differentially accessible peaks were evenly distributed over the genome (Fig. 1e) and annotated to 668 unique genes *(from GRCh38.p13, Table S6)* using the widely used method of proximity to the next TSS. The proportion of protein-coding genes was comparable to that of all measured peaks *(82%, p* > *0.86)*. Interestingly, differentially accessible peaks were significantly enriched in regions containing a known enhancer of gene expression (Fig. 1f & Suppl. Fig. S6g) and in regions localizing to promoters (closed: 32.4% vs. 15.1%, ****p < 0.0001; open: 18.1% vs. 15.1%, *p < *0.05)* (Fig. 1g & Suppl. Fig. S6c), especially differentially less accessible peaks, suggesting a functional relevance for these peaks. Taken together, we identified an ALS-associated epigenetic signature (named ‘*epiChromALS*’) comprised of 729 differentially accessible genomic regions in peripheral PBMCs of ALS patients.

### epiChromALS is found in all major PBMC cell types

The utilization of bulk PBMC samples for sequencing offers several advantages for the interrogation of chromatin accessibility: it is cheaper, faster and less prone to batch effects than sorting the cells by FACS/MACS or than single-cell sequencing. However, bulk sample sequencing is highly sensitive to variations of PBMC cell types between the experimental groups, as often found when comparing disease patients to healthy controls. Therefore, we next investigated the accessibility of epiChromALS in different PBMC cell types.

**Fig. 2:**
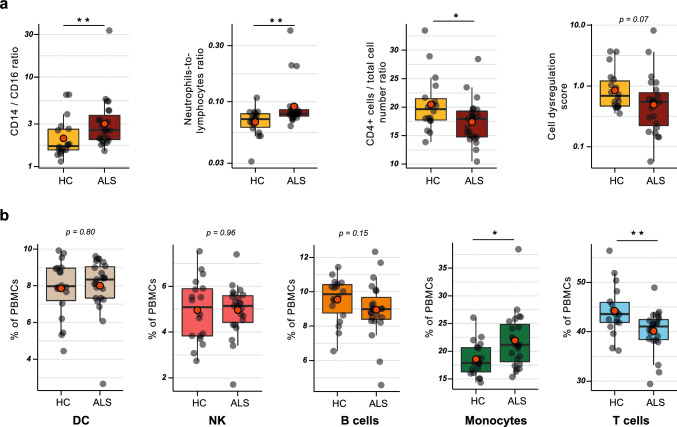
Cell-type dysregulation in ALS PBMCs. **a** Cell-type deconvolution with ABIS shows different cell type ratios which are known to be altered in ALS: These ratios were derived from the cell abundance estimation shown in Suppl. Fig. S8. Cell-type dysregulation score for each person is the average of the weighted absolute z-scores for each cell type in that person, i.e. mean average of *z*-score^2^. **b** Dysregulation of main PBMC subtypes in ALS was estimated with cell deconvolution data and show a significant increase in total monocytes and a decrease in T cells. Boxplots with inter-quartile range, mean average (large orange points) and median (black line). *p < 0.05, **p < 0.01, Mann–Whitney *U*-test

First, we investigated whether cell type composition differs between the ALS patients and the healthy control groups. To this end, we utilized the transcriptome data that we collected from the same samples that were used for the ATAC-seq. We calculated the relative abundance of 12 different cell types found in PBMCs based on their transcriptome signatures with ABIS [55] leading to the estimated cell type abundance expressed in % of total cells (Suppl. Fig. S8). As expected, we found most of the characteristics of ALS PBMCs that we and others have previously demonstrated by flow cytometry: an increased ratio of classical to non-classical monocytes, increased neutrophil-to-lymphocyte ratio, decreased relative abundance of CD4 ^+^ T cells, as well as dysregulated total subcomposition of PBMCs (Fig. 2a) [1, 2, 4, 58, 59]. Furthermore, we observed no change in dendritic cells and natural killer cells in ALS, a slight non-significant decrease in total B cells *(p* = *0.15)*, a significant decrease of total T cells and a significant increase in total monocytes (Fig. 2b).

**Fig. 3:**
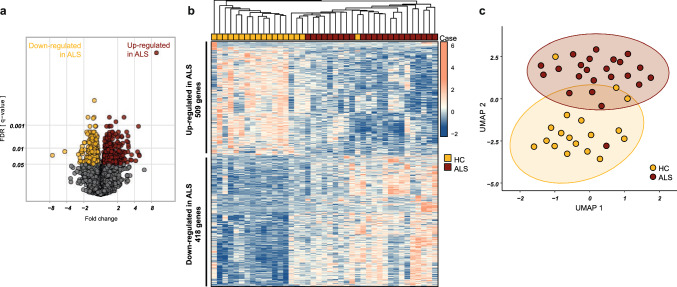
Transcriptome signature of ALS PBMCs. **a**, **b** Differential gene expression analysis in ALS/HC PBMCs. 927 genes were significantly (*q* < *0*.05, FDR) up- or down-regulated in ALS PBMCs and robustly discriminated ALS patients from HCs (WPGMA Average linkage with Euclidean distance). **c** UMAP projection of the sample based on the differentially expressed genes clearly separated HC and ALS sample groups

Since we found a significant variation of cell types between the ALS patients and the healthy controls group, we next asked whether, and to what extent, epiChromALS is driven by this variation. To this end, we compared it to the transcriptomic signature, which is well-known to be influenced by cell type variation [1, 2, 4, 58, 59]. We performed a differential expression analysis on the bulk RNA-seq data from the same PBMC samples and identified an ALS-relevant transcriptomic signature. We detected 30,426 transcripts across all 41 samples, which were expressed at ≥ 1 transcript per million (TPM). Out of these transcripts in the PBMC samples, we found 927 (~ 1.5%) differentially expressed genes at FDR *q* < *0.05* in ALS (Fig. 3a & Table S7). In contrast to epiChromALS, the transcriptomic signature of ALS was more balanced between up-regulated transcripts (*n* = 509, 55%) and down-regulated transcripts (*n* = 418, 45%) and separated HCs from ALS patients more robustly in a supervised hierarchical clustering (Fig. 3b, c & Suppl. Fig. S9). As expected, the differentially expressed genes were enriched in molecular and cellular-function terms related to immune cell function (Suppl. Fig. S10).

**Fig. 4:**
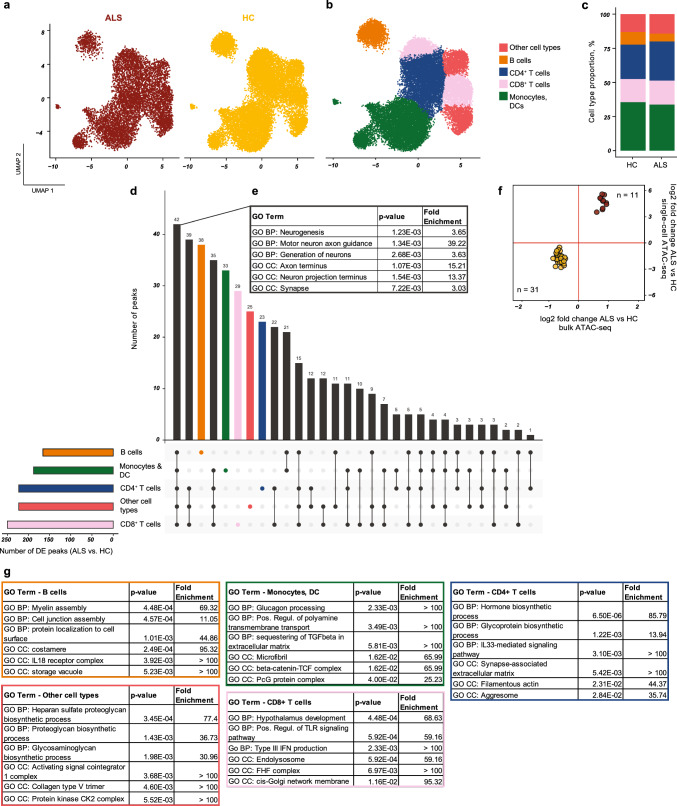
Differentially accessible epiChromALS peaks found in all PBMC cell types map to neuronal genes. Single-cell ATAC-seq analysis of epiChromALS in PBMCs from HC/ALS (*n* = 4/4). **a** UMAP embedding of PBMCs demonstrates successful integration; all cell types were found in HC and in ALS patients’ PBMCs. **b** PBMCs were clustered and 5 major clusters annotated: B cells, CD4^+^ T cells, CD8^+^ T cells, myeloid cells (monocytes + DCs) and other cell types (NK cells, double-negative T cells). **c** Cell type proportions were comparable between HC and ALS patients. **d** UpSet plot demonstrating the number of differentially accessible epiChromALS peaks between HC and ALS in each PBMC cell type. Smaller bar chart: number of differentially accessible peaks in ALS in each cell type; upper bar chart: intersection size for all comparisons. **e** Enrichment of neuronal GO terms in the genes annotated to the 42 peaks that were differentially accessible in ALS in every PBMC cell type. **f** The 42 peaks that were differentially accessible in ALS in every PBMC cell type were 100% concordant in bulk ATAC-seq and single-cell ATAC-seq. **g** GO term enrichment analysis of the epiChromALS peaks that were differentially accessible only in single PBMC cell types. Differential expression analysis with DESeq2 with pseudo-bulk samples for each cell type and each individual (HC/ALS *n* = 4/4), FDR threshold: *q* < 0.05

To test whether the transcriptomic signature of ALS PBMCs is associated with a specific cell cluster, we next investigated its expression in single-cell transcriptomic data of > 152,000 HC PBMCs that are publicly available (Suppl. Fig. S11a; gene expression and cell-surface markers for cell clusters in Suppl. Fig. S12a & b) (GSE164378, GSE100866) [36, 41]. As a negative control, a random gene set of the same size was compared and found to be evenly expressed in all PBMC subtypes (Suppl. Fig. S11b), while the ALS transcriptomic signature was unevenly expressed: up-regulated genes were contributed mostly by classical monocytes, which were increased in the ALS PBMCs, and down-regulated genes were contributed mostly by T cells, which were significantly reduced in ALS (Suppl. Fig. S11c). These data suggest that the differential expression analysis of bulk RNA-seq data is heavily influenced by the cell-type dysregulation of PBMCs in ALS. To test whether epiChromALS gained by bulk ATAC sequencing is also biased by the cell-type dysregulation, the distribution of epiChromALS in publicly available single-cell ATAC-seq data from 14,761 PBMCs from four HCs was investigated (Suppl. Fig. S11d-f) and the corresponding 197,739 chromatin accessibility peaks (10X Genomics) found in them. From the 729 peaks in epiChromALS, we could find 517 peaks in the single-cell ATAC-seq data (71%). Although epiChromALS was less influenced by cell-type dysregulation than the transcriptomic signature, it still showed enrichment in specific cell clusters. Therefore, we next set out to investigate the differential accessibility of epiChromALS between HC and ALS on a cell-type level. We generated single-cell ATAC-seq data in PBMCs from four ALS patients, quantified the accessibility of the 729 epiChromALS peaks, and compared it to the data from HCs (details and statistics of single-cell ATAC-seq in Table S8). To this end, cells were grouped into five major PBMC cell types: ‘B cells’ (B-memory + naïve B cells), ‘CD4^+^ T cells’ (naïve and memory), ‘CD8^+^ T cells’ (naïve and memory), ‘myeloid cells’ (classical monocytes, non-classical monocytes, pDCs and mDCs) and ‘other’ cell types (NK cells and double-negative T cells) and differential accessibility analysis performed with DESeq2 in a pseudo-bulk manner, comparing 4 HC samples to 4 ALS samples for every of the major cell types as recommended before to avoid pseudo-replication [39]. All 729 epiChromALS peaks could be quantified in this analysis. 434 peaks were significantly differentially accessible with a *q* < 0.05 in at least one of the five PBMC cell types (Fig. 4). Interestingly, 42 peaks were significantly differentially accessible in all cell types, suggesting that these peaks result from systemic environmental triggers or from a complex genetic predisposition. These 42 peaks’ differential accessibility was 100% concordant with their differential accessibility in bulk ATAC-seq and they were enriched in neuronal GO-terms, in contrast to peaks that were differentially accessible exclusively in a single cell type, which mapped to immune system-related GO terms.

### epiChromALS is associated with neuronal function and is enriched in neurons and oligodendrocyte precursor cells of ALS brain

**Fig. 5:**
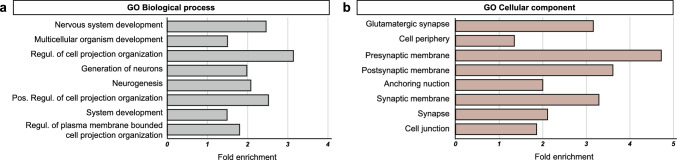
Association of epiChromALS with neuronal function. **a**, **b** Enrichment analysis of epiChromALS using GO for biological processes and cellular components. Bar charts showing the most significant hits together with their fold enrichment. GO terms were associated with neurodevelopmental and synaptic functions. Complete list of GO terms is shown in Suppl. Table S9

In the next step, the functional relevance of epiChromALS was investigated by analyzing the *Gene Ontology* (*‘GO’*) terms associated with the genes annotated to it. In contrast to the transcriptomic ALS signature of PBMCs, which was predominated by general cell physiology and immune cell function GO terms (Suppl. Fig. S10), epiChromALS was strongly associated with GO terms related to neuronal function and neuron differentiation (Fig. 5a-b & Table S9). Some of the most significantly enriched biological processes included *‘nervous system development’*, *‘neurogenesis*’, and *‘generation of neurons’*, while the most significantly enriched cellular component terms were associated with *‘synaptic membrane’*, *‘pre-synaptic membrane’* and *‘post-synaptic membrane´*. Interestingly, the same/similar GO terms were enriched in the 41 epiChromALS peaks, which were differentially accessible in all PBMC cell types (Fig. 4), and thus likely systemic. By contrast, the epiChromALS peaks that were differentially accessible only in a single PBMC cell type were almost exclusively associated with immune cell GO-terms. The driving genes of the respective GO term and the proportion of genes that are likewise specifically expressed in the brain are summarized in Table. S10. The peaks that were associated with these terms were almost exclusively less accessible in ALS, suggesting that neurodevelopmental processes and synaptic signal transduction are impaired on an epigenetic level in ALS. Despite the high proportion of epiChromALS peaks over protein-coding genes (82%) and promoters (29%), a large portion of its associated genes was not expressed in PBMCs (~ 35%), suggesting that these genes are specific to other cell types and tissues. Indeed, analysis of the tissue expression of the not-expressed genes with data from *‘The Human Protein Atlas*’ revealed that the highest proportion (48%) of those are specifically expressed in the CNS, further supporting the relevance of the epiChromALS for CNS development and function. A STRING analysis assigned protein-binding partners to 505 of the 668 unique proteins in the epiChromALS and found significantly more interactions between them than expected (expected number of edges: 158; number of edges: 229; PPI enrichment p-value: ****p < *1* × *10 *^*−7*^*)*, indicating a functional association between them.

**Fig. 6:**
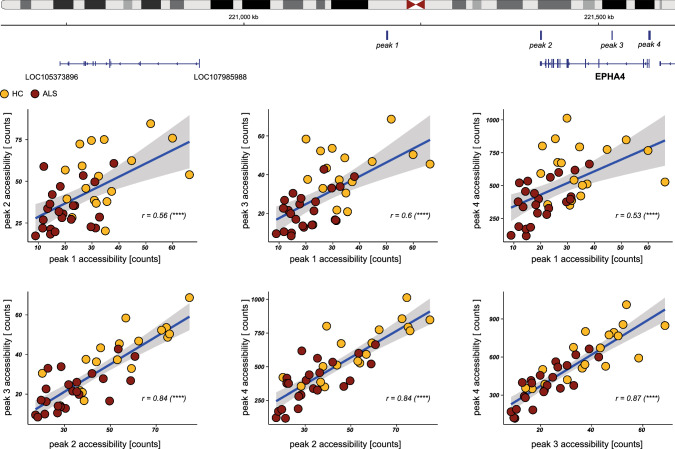
Co-accessibility of epiChromALS peaks in the vicinity of the EPHA4 gene. Four peaks in epiChromALS were located in the proximity (peak 1) or at the gene body (peak 4: promoter; peak 3: intron; peak 2: 3´UTR) of EPHA4. The accessibility of all peaks correlated positively, with the peaks located at the gene body showing the highest correlation (lower row). Spearman’s correlation test

Importantly, enhancers and other genetic elements can act over large genomic distances due to the complex 3D structure of chromatin. In addition, it is well-known that this 3D and functional organization results in a high co-regulation of open chromatin regions, resulting in complex networks of gene regulation by chromatin accessibility. Indeed, co-accessibility analysis of epiChromALS showed high co-accessibility on different scales (Suppl. Fig. S13). Most of the peaks were correlated positively, while some were correlated negatively. For example, four peaks on chromosome 2 were mapped to the EPHA4 gene, which plays an important role in neurodevelopmental processes and has previously been associated with ALS. The accessibility of all these peaks was strongly correlated, with the distal peak showing the least association (Fig. 6). Two other epiChromALS peaks, which mapped to the MYO5B gene were significantly negatively correlated (Suppl. Fig. S14), demonstrating that the co-regulation of accessible chromatin regions results from specific cellular or molecular processes rather than from technical or experimental bias. Thus, regulation of gene expression by chromatin accessibility occurs on different genomic scales and simple annotation of peaks to the closest gene does not reflect the full impact of a single chromatin peak. To control for such bias, we expanded the annotation of epiChromALS peaks by correlating their accessibility to the expression of genes within a 500 kb window from both sides of a peak using two different methods: correlation of the bulk ATAC-seq data to the matched bulk RNA-seq data, where every observation point is an individual (HC/ALS patient), and correlation of single-cell ATAC-seq data to RNA-seq data from the same cell in a public dataset of Multiomic ATAC-seq + Gene expression in one HC (10X Genomics), where each observation point is a cell/metacell*.* Using this approach, 539 additional genes could be added to epiChromALS; however, the functional enrichment of neuronal terms remained similar, further confirming the robust association of the epiChromALS with neuronal function (Tables. S9 & S11–12). The total of 1207 genes and the methods used to annotate them are listed in Table S11 and Fig. S15; the exact annotation of each epiChromALS peak along with statistics from the differential accessibility analysis in Table S13).

**Fig. 7:**
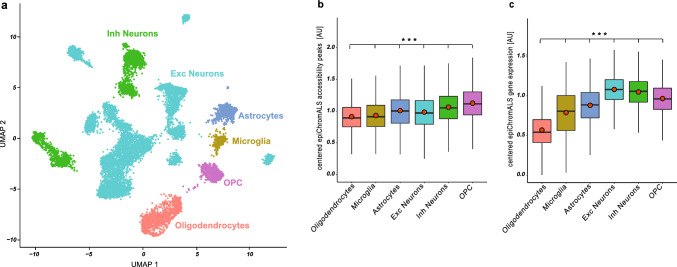
Integration of epiChromALS and single-cell ATAC seq of ALS brain. **a** UMAP projection of identified cell types by single-cell ATAC-seq of human ALS motor cortices. **b**, **c** Averaged accessibility of the epiChromALS peaks (265/729) and averaged predicted expression of the epiChromALS genes (437/668) in different motor cortex cell types. Boxplots: median ± inter-quartile range with mean average (orange dots). Each subpopulation expression was significant to any other subpopulations in (**c**, **d**) (***p < 0.001)

As the functional analysis of epiChromALS strongly suggested association to neuronal function, we next investigated if the epiChromALS genomic regions can be found in the CNS or are even enriched in specific CNS cell types. To this end, we performed single-cell ATAC-seq of > 11,200 nuclei *(‘snATAC-seq’)* purified from the post-mortem motor cortex *(M4)* of 3 ALS patients (Fig. 7a, Suppl. Fig. S16) and annotated them to 6 broad cell types *(oligodendrocytes, OPCs, astrocytes, microglia, inhibitory neurons and excitatory neurons*) (Suppl. Fig. S12c). 265/729 peaks from epiChromALS (36%) were also found in the CNS cells. Due to this low number of overlapping genes, we looked for the genes that are associated with those peaks and checked their predicted expression in the different brain cell types. Here we found that 437 of the 668 epiChromALS genes (65%) were expressed in CNS cells. In both comparisons, epiChromALS peaks or genes were specifically enriched in neurons and oligodendrocyte precursor cells (‘OPCs’) (Fig. 7b, c), further suggesting its relevance for neuron generation and function.

### epiChromALS is enriched in genes previously associated with ALS

**Fig. 8:**
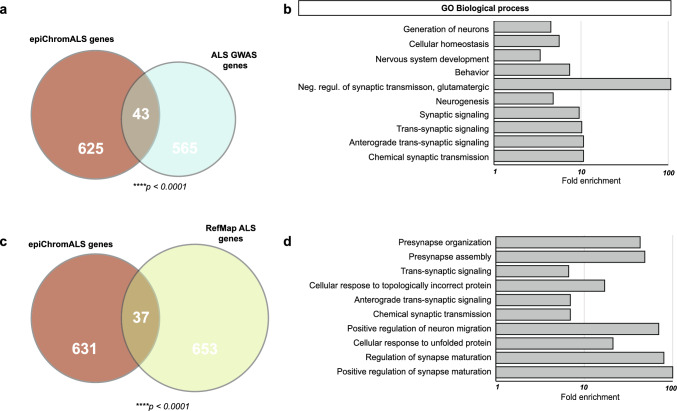
Association of epiChromALS with ALS genes. **a** The Venn diagram represents the genes from the overlap between epiChromALS and the GWAS SNPs study. **b** GO enrichment analysis of the overlapped 43 genes demonstrates that they are associated with neuronal function. Bar charts show the top 10 terms for biological processes, ranked by raw *p*-value. **c** Overlap between epiChromALS and RefMapALS identified 37 common genes. **d** GO enrichment analysis of these 37 genes demonstrates these are associated with synaptic function. A detailed list of the overlapped genes is shown in Tables S13 & S14

To explore the association of genes annotated to epiChromALS with the disease etiology, we compared them to genes with known ALS GWAS association in the GWASdb SNP-Disease Associations database [48]; https://maayanlab.cloud/Harmonizome/gene_set/amyotrophic+lateral+sclerosis/GWASdb+SNP-Disease+Associations]. Out of 608 GWAS ALS genes, 43 were also found in epiChromALS, showing a significant threefold enrichment over a stochastically expected overlap (which would respond to ~ 12 genes) (****p < *0.0001, OR* = *3.0 (2.1–3.1), Fisher’s exact test)* (Fig. 8a & Table S14), while controls with randomly sampled genes from all genes annotated to peaks in the study showed no enrichment, as expected. Interestingly, the 43 common genes were enriched in synaptic GO-terms (Fig. 8b) and had highly significant enrichment of interactions between them (STRING: expected number of edges: 5; number of edges: 28; PPI enrichment p-value: ****p < *1* × *10*^*–11*^*)*, while random samples of 43 peaks from either all GWAS ALS genes or from all epiChromALS peaks showed no functional enrichment, suggesting a mechanistic link between these genes, neuronal development/function, and epigenetic mechanisms.

Recently, machine-learning strategies have been applied to reveal associations of novel genes with ALS [60] by combining GWAS data with functional genomics. These ALS-associated genes termed *‘RefMap ALS genes´* shared with epiChromALS the association to axonal and synaptic GO terms. We therefore next investigated whether some of the RefMap genes can be found also in epiChromALS. Indeed, RefMap (690) genes and epiChromALS (668) genes showed a > twofold, statistically significant overlap of 37 genes *(OR* = 2.3 (1.6–3.2), ****p < *0.0001, Fisher’s exact test)* (Fig. 8c & Table S15) and the genes which could be found in both gene sets were enriched in synaptic GO terms (Fig. 8d). Moreover, there was a significant overlap also between curated ALS genes from the same study [60, 61] and epiChromALS *(OR* = 2.3 (1.3–3.9), **p < *0.01, Fisher’s exact test)*, including *OPTN* and 14 additional manually curated ALS genes.

### epiChromALS correlates with the age of disease onset

**Fig. 9:**
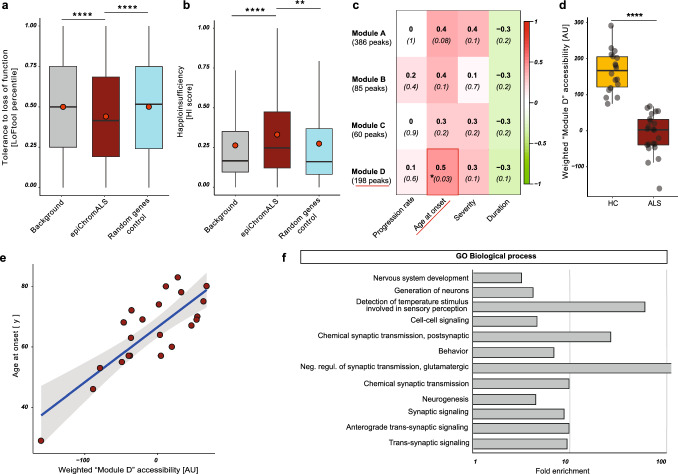
Association of epiChromALS with ALS. **a** epiChromALS genes show increased susceptibility to loss of function and haploinsufficiency (**b**). **p < 0.01, ****p < 0.0001, Fisher ‘s exact test and Mann–Whitney *U*-test. (**c**) WGCNA of epiChromALS accessibility in ALS patients. The strongest association was found with the age of disease onset. **d**, **e** A lower weighted accessibility of the WGCNA module with the strongest association to the age of onset discriminates robustly HCs from ALS patients and predicts an earlier age of onset. ****p > 0.0001, Mann–Whitney *U*-Test. **f** GO enrichment analysis of those genes that were summarized in Module D

Next, we investigated the epiChromALS genes for susceptibility to haploinsufficiency and loss of function [62, 63]. The epiChromALS genes were both significantly prone to loss-of-function (lower percentile in the LoFtool score, Fig. 9a) and haploinsufficiency (higher HI score, Fig. 9b). Of note, genes that score high for haploinsufficiency and loss-of-function are often associated with modulation of the age of onset and mechanisms of early-onset-disease [60, 64]. Therefore, we next investigated if epiChromALS is correlated with disease characteristics by WGCNA (Weighted-Gene Co-expression Network Analysis) of the 729 epiChromALS peaks. WGCNA could identify four clusters (‘modules’) of peaks that were similarly co-accessible in the ALS patients’ samples. Correlation analysis of the block eigenvalues with the clinical disease parameters ‘age at onset’, ‘disease duration’, ‘disease severity’ and ‘disease progression rate’ identified the strongest correlation of the chromatin accessibility signature with the age of disease onset (Fig. 9c). Due to the relatively short disease course, age of onset was highly correlated with the age of ALS patients; however, the accessibility of epiChromALS was not significantly correlated to age in the HC group, suggesting that the association is specific to the age of onset of disease and not generally to age. Of all 4 blocks of peaks, a block of 198 peaks (‘Module D’) showed the strongest association with age of onset (Pearson’s correlation coefficient: 0.5, *p < 0.05). The weighted module accessibility of this module was strongly decreased in ALS patients (Fig. 9d) and correlated strongly with the age of onset (Fig. 9e). Interestingly, the 198 genes in this module were again enriched in synaptic GO terms, highlighting a possible mechanistic link between the ALS chromatin signature and disease etiology (Fig. 9f).

## Discussion

The present study demonstrates the presence of a genome-wide epigenetic signature of ALS *(‘epiChromALS’)* detectable in the chromatin accessibility of patients with sporadic ALS. By combining transcriptome (RNA-seq) and chromatin accessibility (ATAC-seq) interrogation from the same sample, epiChromALS is shown to be less influenced by typical confounding factors in peripheral blood cells like cell-type variations and overrepresentation of cell-type specific transcripts. Furthermore, epiChromALS is associated with neuronal terms. Single-cell sequencing of peripheral blood cells and ALS motor cortex as well as systems biology approaches thoroughly integrating our data sets underline the disease relevance of epiChromALS. Moreover, our study is the first to link epigenetic marks of neurodegenerative disease with physiological relevance to the affected tissues in the CNS and in the periphery, suggesting that they: i) can originate from impacts like environmental stimuli and/or genetic predisposition and ii) can be used to study disease mechanisms.

Chromatin accessibility results from the cumulative effect of different epigenetic mechanisms: histone modifications, DNA-methylation and nucleosome positioning [65–69]. These modifications can result from the local milieu of a cell population, thus affecting only a specific population of cells, as is the case in epigenetic mechanisms of cell differentiation [70–72]; or they can result from systemic triggers like environmental impacts [73] and genetic polymorphism [74] and thus affect many different cell populations across different tissues and be inherited in daughter cells after division. Thus, we hypothesized that if environmental influences and/or genetic predisposition result in disease-associated chromatin accessibility changes, some of these should be detectable peripherally, e.g. in peripheral blood cells. Indeed, employing single-cell sequencing, we observed that some disease-specific chromatin changes were found in most/all PBMC cell types, while others were specific to different cell types and thus probably local. The inheritance of chromatin accessibility changes across cell divisions has been previously suggested to be maintained by the differential activity of transcription factors, which could explain how systemic disease-related chromatin changes are maintained in the constantly renewing blood cells [75]. In addition, if systemic environmental triggers are persisting, it is possible that these result in the continued de novo generation of the same epigenetic changes with every new cell generation.

Multiple lines of evidence support the idea of an association of the chromatin accessibility signature with ALS: epiChromALS is highly enriched in genes previously associated with ALS based on GWAS data [48] and significantly overlap with ‘RefMapALS genes’ that have recently been described as ALS-associated genes by integration of functional genomics with GWAS summary statistics [60]. Furthermore, epiChromALS genes were both significantly haploinsufficient and prone to loss-of-function, features that are often associated with modulation of the age of onset of diseases [60, 64]. Indeed, splitting epiChromALS into four blocks and reducing them to a single block score by WGCNA analysis demonstrated that a portion of epiChromALS strongly correlates with the age of ALS disease onset. Previously found specific epigenetic marks, e.g. DNA methylation marks have also been associated with ALS disease onset [10]. Together, epigenetic alterations seem to highly affect ALS disease age of onset. However, our observations do not exclude the possibility that epiChromALS is associated also to other neurodegenerative diseases. Although the association of epiChromALS with the age of onset of ALS was stronger than with age, both factors cannot be reliably dissociated in ALS. It is therefore possible that epiChromALS is a more general feature not specifically of ALS but of (accelerated) aging. To investigate this, the signature has to be compared to the ATAC profiles of other neurodegenerative disease that are highly associated with aging, like Alzheimer’s disease and Parkinson’s disease.

Genes annotated to epiChromALS were enriched for GO terms *‘nervous system development’*, *‘neurogenesis*’ and *‘generation of neurons’*. The main drivers of these enrichments include EPHA4, EPHA3, NRXN3 and ANK3. These genes are all involved in neuron differentiation and development, as well as cell generation of neurons.

A challenge in using multi-omic datasets is understanding how the direction of a change impacts disease pathogenesis. While we clearly cannot state whether the observed changes in chromatin accessibility in ALS are conductive to the course of the disease or a cellular attempt at a homeostatic response to physiological insults, we identify blood cells recapitulating epigenetic alterations, thus providing insight into potentially involved mechanisms in the brain. In contrast to the RNA signature obtained from PBMCs from ALS patients that was not associated with neuronal networks, epiChromALS was very strongly associated with neuronal terms. Moreover, the most significant differential peaks in ALS PBMCs were almost completely driven by inaccessible regions, suggesting that the respective annotated genes are linked to impaired functions. Most importantly, epiChromALS is highly enriched in neurons and OPCs from the motoric cortex from ALS patients. Most of the respective annotated genes provided by epiChromALS are not expressed in blood cells, thus conventional transcriptomics analysis alone would not have discovered a CNS-specific signature. Moreover, this finding points towards a global alteration that is detrimental in cells expression of the respective genes is needed, whereas in PBMCs they are a concomitant without consequences.

As recently outlined [76], sampling of affected tissues is not feasible in neurodegenerative diseases, where it is associated with high-risk, highly invasive manipulations. Scalable sampling with the potential for longitudinal follow-up and minimal burden for the patients is crucial for the identification of pathological mechanisms and their monitoring during therapeutic approaches. We suggest that this need could be at least partially addressed by studying the chromatin accessibility of PBMCs. If the chromatin changes in ALS PBMCs occur prior to disease onset, the inclusion of asymptomatic carriers of fALS mutations in longitudinal studies of epiChromALS contributes to better monitoring of the involvement of chromatin remodeling in the development of the disease. Further, this provides new insights into a potential epigenetic predisposition that may exist in addition to a genetic preload. Finally, observational studies can use these alterations to identify individuals at high risk for ALS and include them in prevention studies, as the disease progresses rapidly after the disease onset.

An importation limitation of the present study is the small sample size in the comparison of epiChromALS with the chromatin accessibility in the human motor cortex and has to be considered for the interpretation of the data. This also restricts linking upper vs lower motor disease onset to epiChromALS. Further studies including larger sample sizes and the comparison of epigenetic profiles in different brain regions as well as spinal cord tissue is of utter need. In addition, studies of the epigenetic regulation of gene expression imply a holistic approach where affected pathways and systems are identified rather than isolated genetic elements. Indeed, the limitation of genetic studies to isolated genetic elements has been proposed to account for the missing heritability observed in ALS [77, 78].

In conclusion, our data suggest that chromatin accessibility, resulting from genetic predisposition and/or epigenetic regulation, is associated with ALS and can be reflected in blood cells.

## Supplementary Information

Below is the link to the electronic supplementary material.

Supplementary file1 (PDF 2702 KB)

Supplementary file2 (XLSX 307 KB)

## Data Availability

The processed sequencing data generated in this study are available from the corresponding author upon reasonable request.

## Abbreviations

ALS: Amyotrophic Lateral Sclerosis
ATAC-seq: Assay for transposase-accessible chromatin using sequencing
CNS: Central nervous system
epiChromALS: Epigenetic chromatin accessibility signature in ALS
FDR: False discovery rate
FE: Fold enrichment
GO: Gene ontology
HC: Healthy control
mDC: Myeloid dendritic cells
NK: Natural killer cells
OPC: Oligodendrocyte progenitor cell
PBMC: Peripheral blood mononuclear cell
pDC: Plasmacytoid dendritic cells
snATAC-seq: Single-nuclei assay for transposase-accessible chromatin using sequencing
TAD: Transcriptionally associated domain
TPM: Transcripts per million
TSS: Transcription start site
WGCNA: Weighted-Gene Co-expression Network Analysis
