# Supplementary material for: ALS is imprinted in the chromatin accessibility of blood cells
Source: Cell Mol Life Sci. 2023 Apr 24;80(5):131. doi: 10.1007/s00018-023-04769-w (PMC10126052; doi:10.1007/s00018-023-04769-w)

ALS is Imprinted in the Chromatin Accessibility of Blood Cells

Julia K. Kühlwein[1], Wolfgang P. Ruf[1], Katharina Kandler[1], Simon Witzel[1], Christina Lang[1], Medhanie A. Mulaw[2], Arif B. Ekici[3], Jochen H. Weishaupt[4], Albert C. Ludolph[1, 5], Veselin Grozdanov[1, □], Karin M. Danzer[1, 5, θ *]

[1] Department of Neurology, University Clinic, University of Ulm, Ulm, Baden-Wuerttemberg, 89081, Germany
[2] Medical Faculty, University of Ulm, Ulm, Baden-Wuerttemberg, 89081, Germany
[3] Institute of Human Genetics, University Clinic Erlangen, Friedrich-Alexander-University Erlangen-Nürnberg, Erlangen, Bayern, 91054, Germany
[4] Division for Neurodegenerative Diseases, Neurology Department, University Medicine Mannheim, Heidelberg University, Mannheim, Baden-Wuerttemberg, 68167, Germany
[5] German Center for Neurodegenerative Diseases (DZNE), Ulm, Baden-Wuerttemberg, 89081, Germany
[θ]These authors contributed equally.

*Correspondence:
Prof. Dr. Karin M. Danzer
karin.danzer@dzne.de

Supplementary Figures

Table of Contents

Supplementary Fig. S1: Bulk ATAC-seq quality control statistics of PBMC samples.

Supplementary Fig. S2: Fragment profiles of bulk ATAC-seq from PBMCs.

Supplementary Fig. S3: TSS profile of bulk ATAC-seq from PBMCs.

Supplementary Fig. S4: Bulk ATAC-seq peak statistics.

Supplementary Fig. S5: Quality control parameters of ATAC-seq PBMCs.

Supplementary Fig. S6: Comparison of differential chromatin accessibility in ALS vs. HC and a random permutation groups control.

Supplementary Fig. S7: PCA analysis of ALS and HC sample with ATAC peaks

Supplementary Fig. S8: Relative cell type abundance in the transcriptome signature of ALS and HC PBMCs.

Supplementary Fig. S9: PCA analysis of ALS and HC sample with gene expression data.

Supplementary Fig. S10: Functional analysis of differentially expressed genes in PBMCs of ALS patients and HCs.

Supplementary Fig. S11: Cell-type contribution to the transcriptome signature of ALS PBMCs and epiChromALS signature.

Supplementary Fig. S12: Cell marker of single-cell experiments.

Supplementary Fig. S13: epiChromALS co-accessibility.

Supplementary Fig. S14: Co-accessibility of the two ATAC peaks in epiChromALS that were associated with the MYO5B gene.

Supplementary Fig. S15: Different annotation methods for epiChromALS.

Supplementary Fig. S16: Quality control parameters of single-cell ATAC-seq of human ALS brains.

Supplementary Figure S1: Bulk ATAC-seq quality control statistics of PBMC samples. Box plots with interquartile range for (**a**) the fraction of reads on a peak, (**b**) TSS enrichment and (**c**) ratio of nucleosome-free to mono-nucleosome fraction. Yellow dots represents healthy controls and red dots ALS patients. The red line in (a) and (b) shows the ENCODE threshold. The defined threshold for the fraction of reads in called peak regions should be >0.3, though values greater than 0.2 are acceptable. For the TSS annotation values of 5-7 are defined as acceptable and >7 as ideal.

Supplementary Figure S2: Fragment profiles of bulk ATAC-seq from PBMCs. (**a**) Representative Tape Station fragment size distribution after library construction. The first peaks shows the nucleosome-free fragments, the second the mono-nucleosomes and the third the di-nucleosomes. (**b**) Representative sample for the ATAC-seq fragment length distribution. Graph shows periodicity signal of nucleosomes. (**c**) Comparison of ATAC-seq fragment length distribution from 18 HCs (in yellow) and 23 ALS patient PBMCs
(in red). Bandwidth for line smoothing: 10 bp.

Supplementary Figure S3: TSS profile of bulk ATAC-seq from PBMC. Heatmap shows the correlation of a representative ATAC peaks in a ± 2kb window around the TSS after alignment and filtering.

Supplementary Figure S4: Bulk ATAC-seq peak statistics. (**a**) Distribution of peak width, (**b**) distribution of peaks over chromosomes (autosomes + sex chromosomes) and (**c**) number of peaks assigned per gene of bulk ATAC-seq PBMCs from ALS patients and HCs. Violin plots (distribution) with boxplot inlets (interquartile range) and mean average (orange points).

Supplementary Figure S5: Quality control parameters of ATAC-seq PBMCs. (**a-d**) Normalized total filtered reads, distinct fragments, number of peaks and peaks per sequencing depth were comparable between ALS and HC.

Supplementary Figure S6: Comparison of differential chromatin accessibility in ALS vs. HC and a random permutation groups control. (**a,d**) Quantile-quantile plots of expected vs. observed p-values for both comparisons. (**b, e**) Volcano plots of differentially chromatin accessibility peaks for either epiChromALS or a random set of differentially accessible peaks. (**c, f**) Proportions of less accessible (‘closed’) and more accessible (‘open’) peaks in all HC vs. ALS / Random1 vs. Random2 comparisons and in significant hits. (**g**) Differentially accessible genomic regions in ALS vs. HC, but not in Random1 vs Random2 are enriched in enhancers.

Supplementary Figure S7: PCA analysis of ALS and HC sample with ATAC peaks. Distribution of HC and ALS ATAC samples in a low-dimensional space with using the normalized, scaled counts of all >760,000 ATAC peaks ((**a**) PCA and (**b**) UMAP), and using the scaled, normalized counts of the 729 epiChromALS ATAC peaks ((**c**), PCA).

Supplementary Figure S8: Relative cell type abundance in the transcriptome signature of ALS and HC PBMCs. Estimated cell-type abundance in ALS and HC PBMCs was calculated with ABIS and identified 12 different PBMC subpopulations. In this analysis, only classical monocytes were significantly enriched. Boxplots with interquartile range. ALS patients are shown in red and healthy controls in yellow. **p<0.01, Mann-Whitney U-test.

Supplementary Figure S9: PCA analysis of ALS and HC sample with gene expression data. Distribution of HC and ALS RNA samples in a low-dimensional space with using the normalized, scaled counts of all >60 000 genes ((**a**) PCA and (**b**) UMAP), and using the scaled, normalized counts of the 927 differentially expressed genes between ALS and HC samples ((**c**) PCA).

Supplementary Figure S10: Functional analysis of differentially expressed genes in PBMCs of ALS patients and HCs. (**a-c**) GO enrichment analysis of 927 differentially expressed genes for biological processes, reactome pathways

and cellular components, respectively. Shown are the top 10 GO terms that were filtered for the greatest fold enrichment.

Supplementary Figure S11: Cell-type contribution to the transcriptome signature of ALS PBMCs and epiChromALS signature. (**a**) UMAP projection of cell types identified in single-cell RNA-seq of PBMCs and the expression pattern of the 927 genes differentially expressed in a random set of genes (**b**) or ALS (**c**) in the different cell types. (**d**) UMAP projection of cell types identified in single-cell ATAC se of HC PBMCs. (**e-f**) Accessibility score of the epiChromALS and a random peak control signature in the different subtypes. The random control signature is comparably accessible in all cell types. epiChromALS is highly enriched in B cells and DCs, although these were only slightly dysregulated in ALS and is not enriched in T cells, although these were strongly dysregulated in ALS.

Supplementary Figure S12: Cell Markers of single-cell experiments. (**a**) Cell Surface Markers for the analysis of scRNA PBMCs, (**b**) Gene expression markers for single-cell RNA analysis of PBMCs and (**c**) cell-type markers in the single-cell ATAC-seq analysis of motor cortex. Shown are the normalized expression levels and expression percentage of the expressed genes. Colors represents normalized expression level, and dot size represents the percentage of cells expressing the respective marker gene.

Supplementary Figure S13: epiChromALS co-accessibility. The accessibility of each epiChromALS peak (729 peaks, counts normalized to total effective library size) was correlated to the accessibility of all other epiChromALS peaks. Correlation coefficients (Spearman's ρ) < -0.5 are displayed in shades of blue and > 0.5 in shades of red. Peaks are plotted along the x-axis.

Supplementary Figure S14: Co-accessibility of the two ATAC peaks in epiChromALS that were associated with the MYO5B gene. Top track: Chromosome 18 map with the region that is enlarged (red band). Middle track: the two peaks in epiChromALS in this region: 'peak 1' in intron 5 and 'peak 2' over the MYO5B promoter. Bottom track: a MYO5B transcript map from RefSeq. The accessibility of both peaks was negatively correlated (Spearman's ρ: -0.44 (95% CI: -0.66-0.15), ***p*<0.01).

Supplementary Figure S15: Different annotation methods for epiChromALS. The Venn diagram shows the number of identified genes for each annotation method: blue=annotation of peaks by proximity to the nearest gene upstream or downstream of a peak, red=annotation of peaks by correlating the accessibility in each bulk sample to the RNA-expression in each bulk sample and green = correlation of ATAC accessibility of each peak in single-cell with the RNA-expression profiles in the same cell using the public single-cell multiomic data (10X Genomics).

Supplementary Figure S16: Quality control parameters of single-cell ATAC-seq of human ALS brain. (**a-d**) Fraction of reads in peaks, number of peak fragments, TSS enrichment and nucleosome signal for single-cell ATAC-seq of motor cortex from four ALS patients.

Supplementary Figure S1.

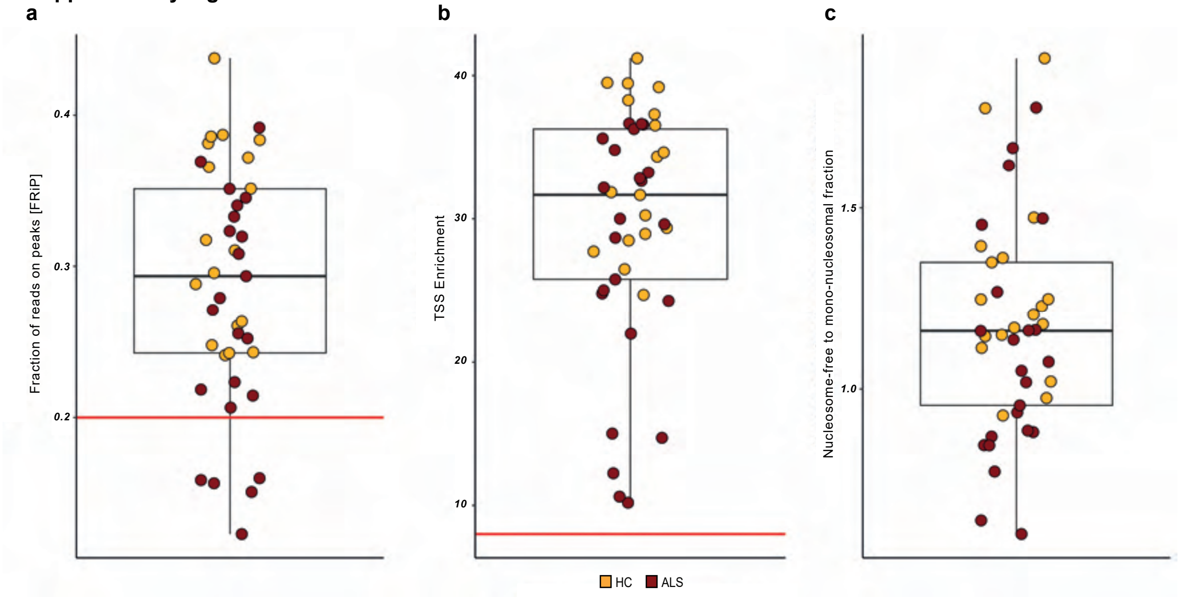

Supplementary Figure S2.

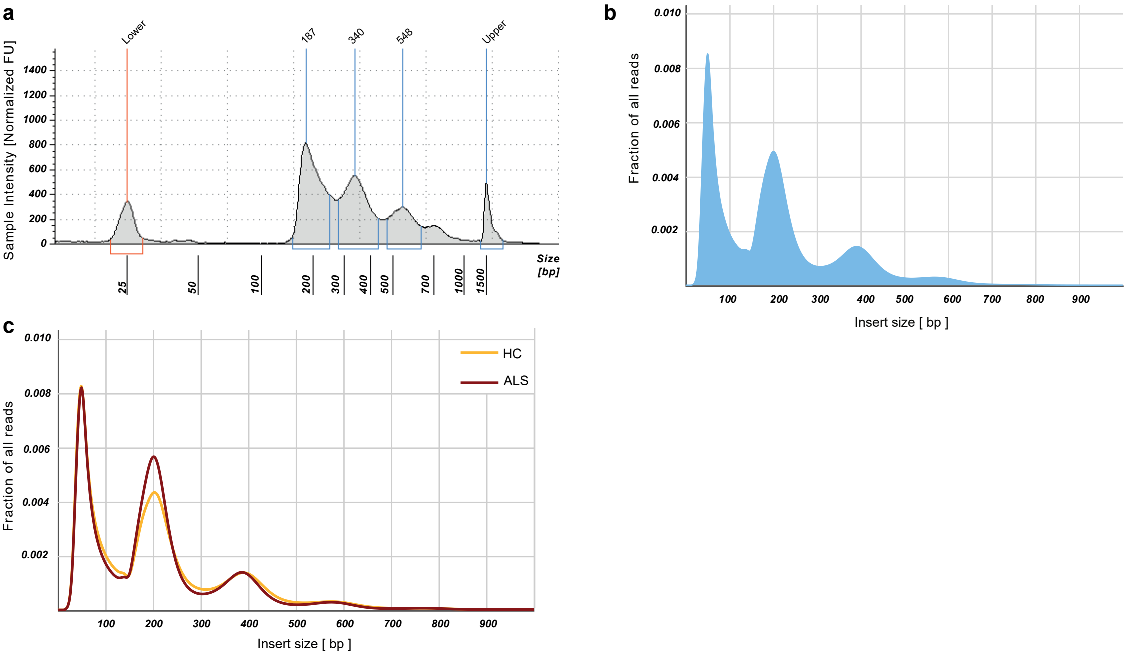

Supplementary Figure S3.

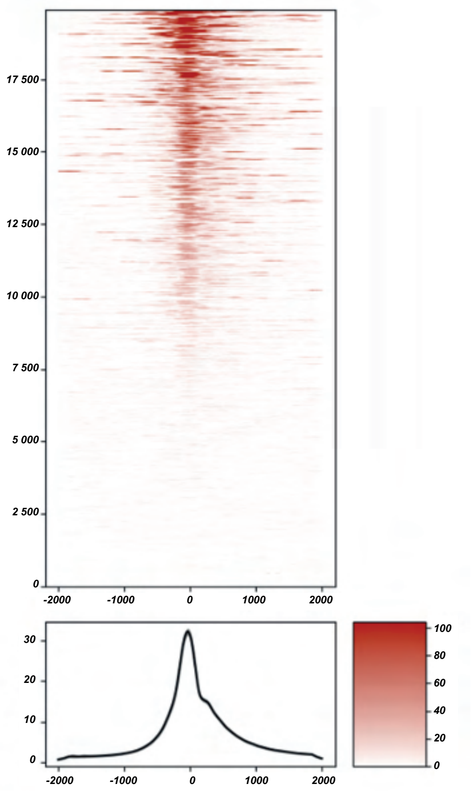

Supplementary Figure S4.

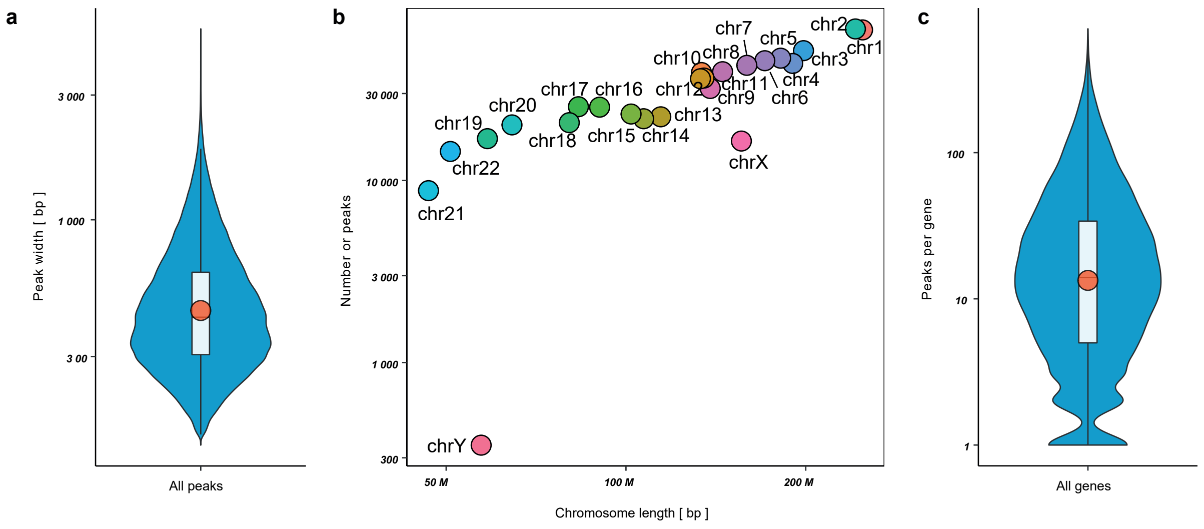

Supplementary Figure S5.

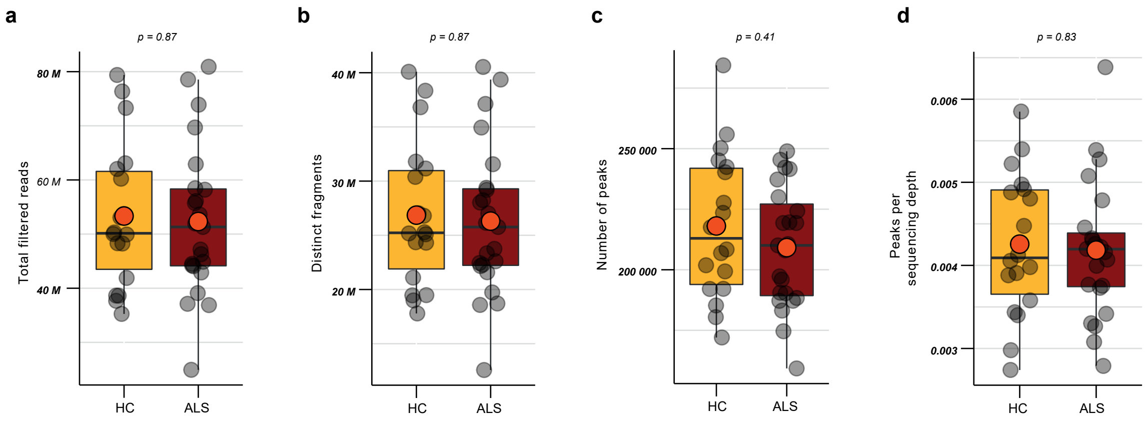

Supplementary Figure S6.

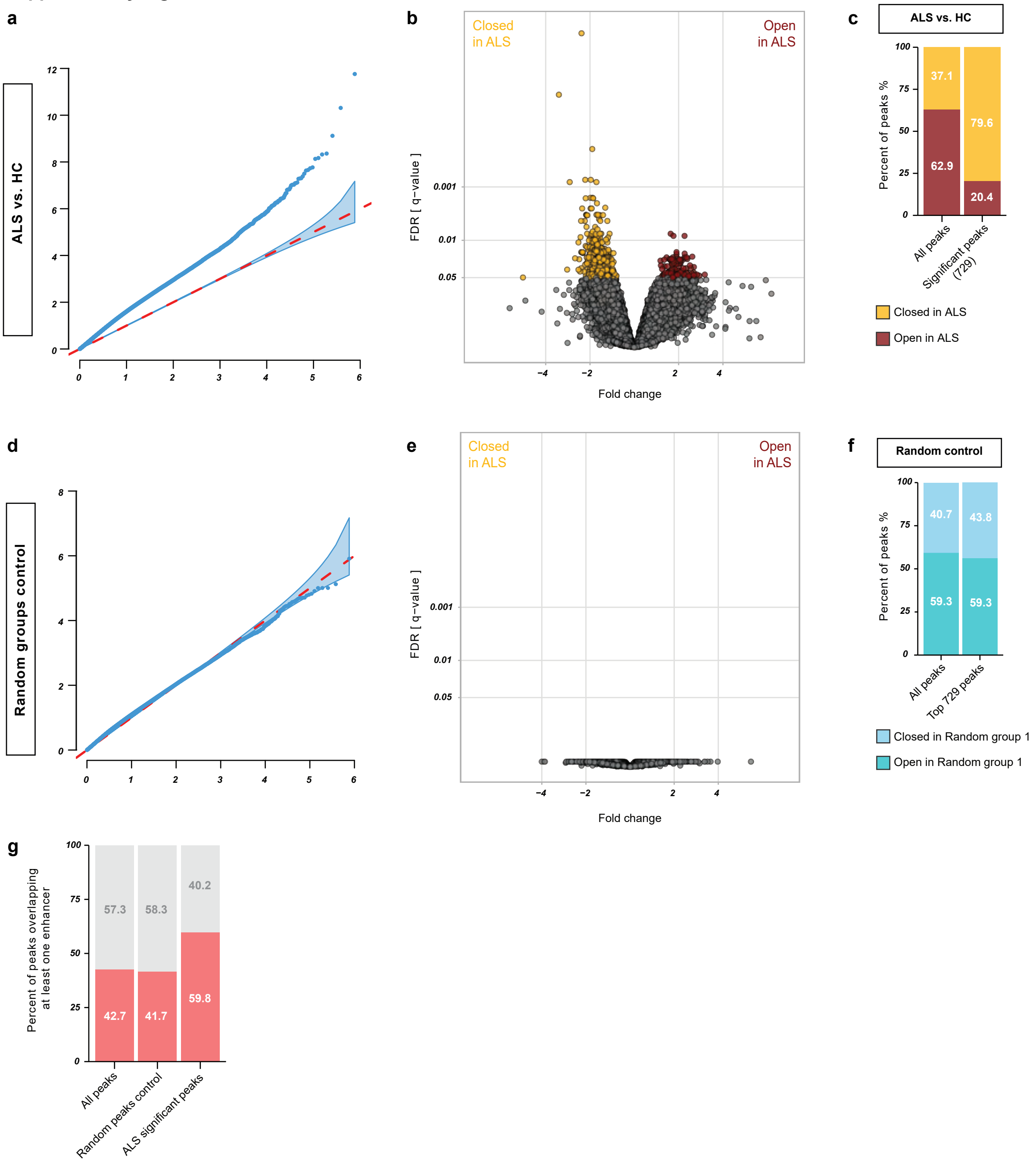

Supplementary Figure S7.

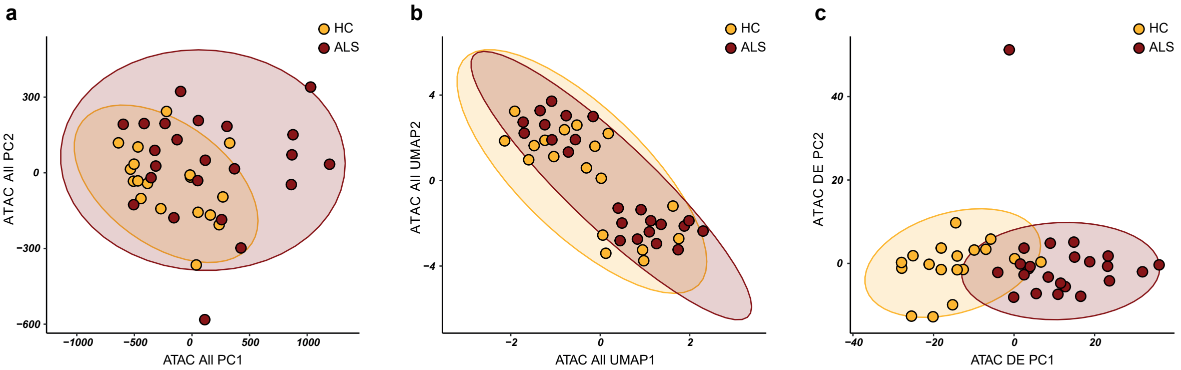

Supplementary Figure S8.

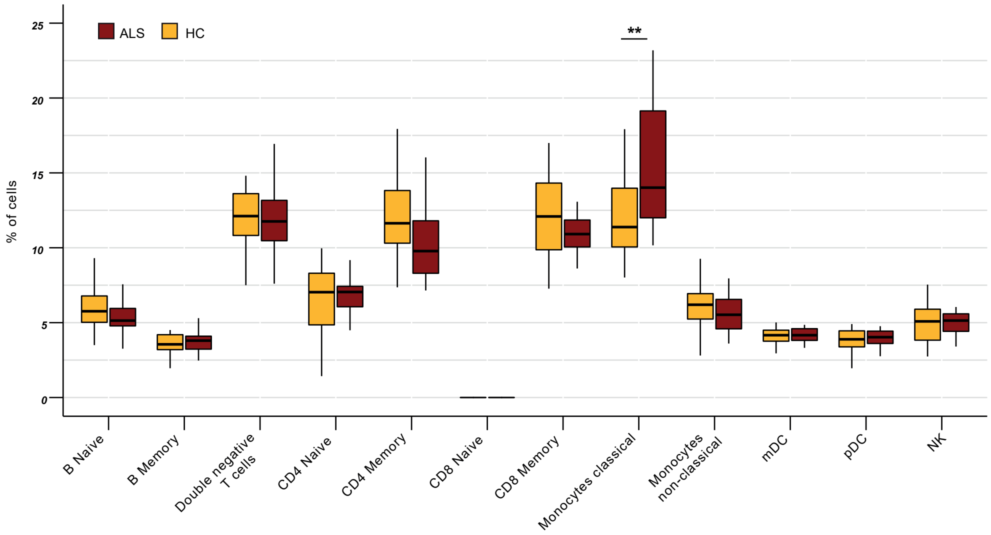

Supplementary Figure S9.

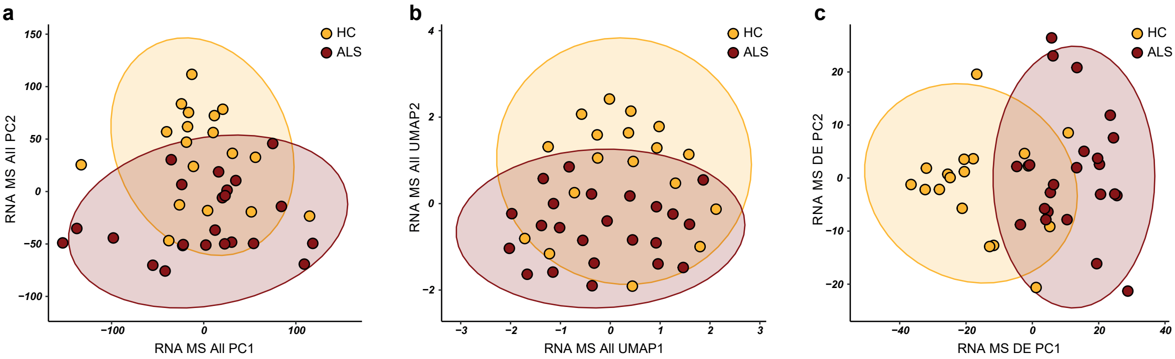

Supplementary Figure S10.

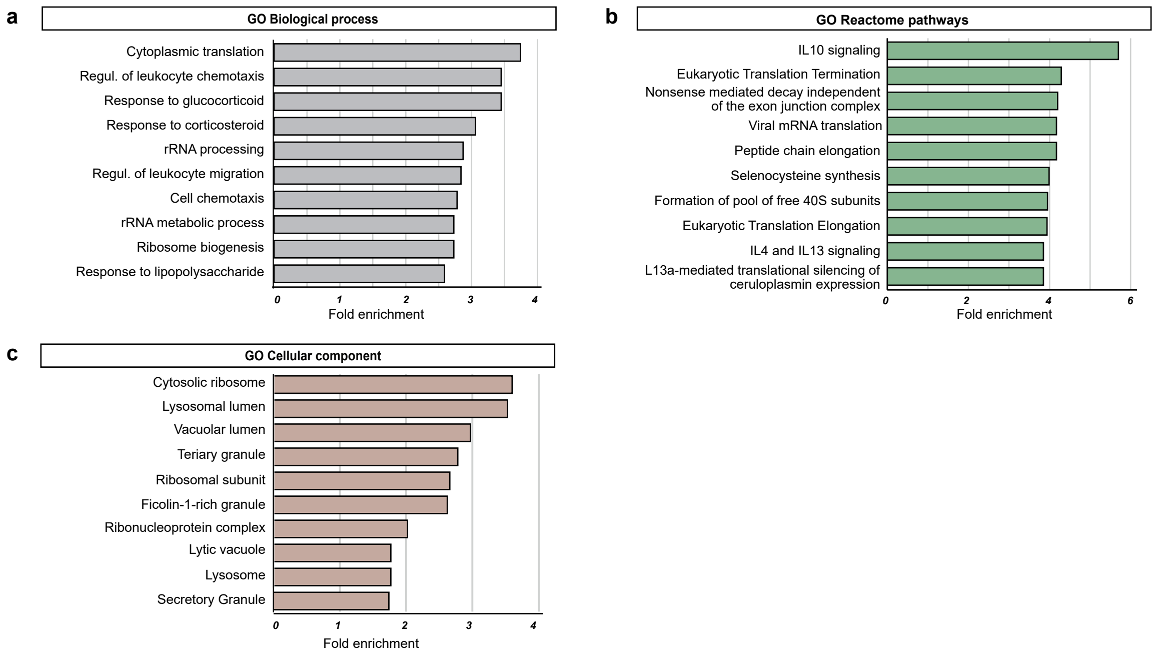

Supplementary Figure S11.

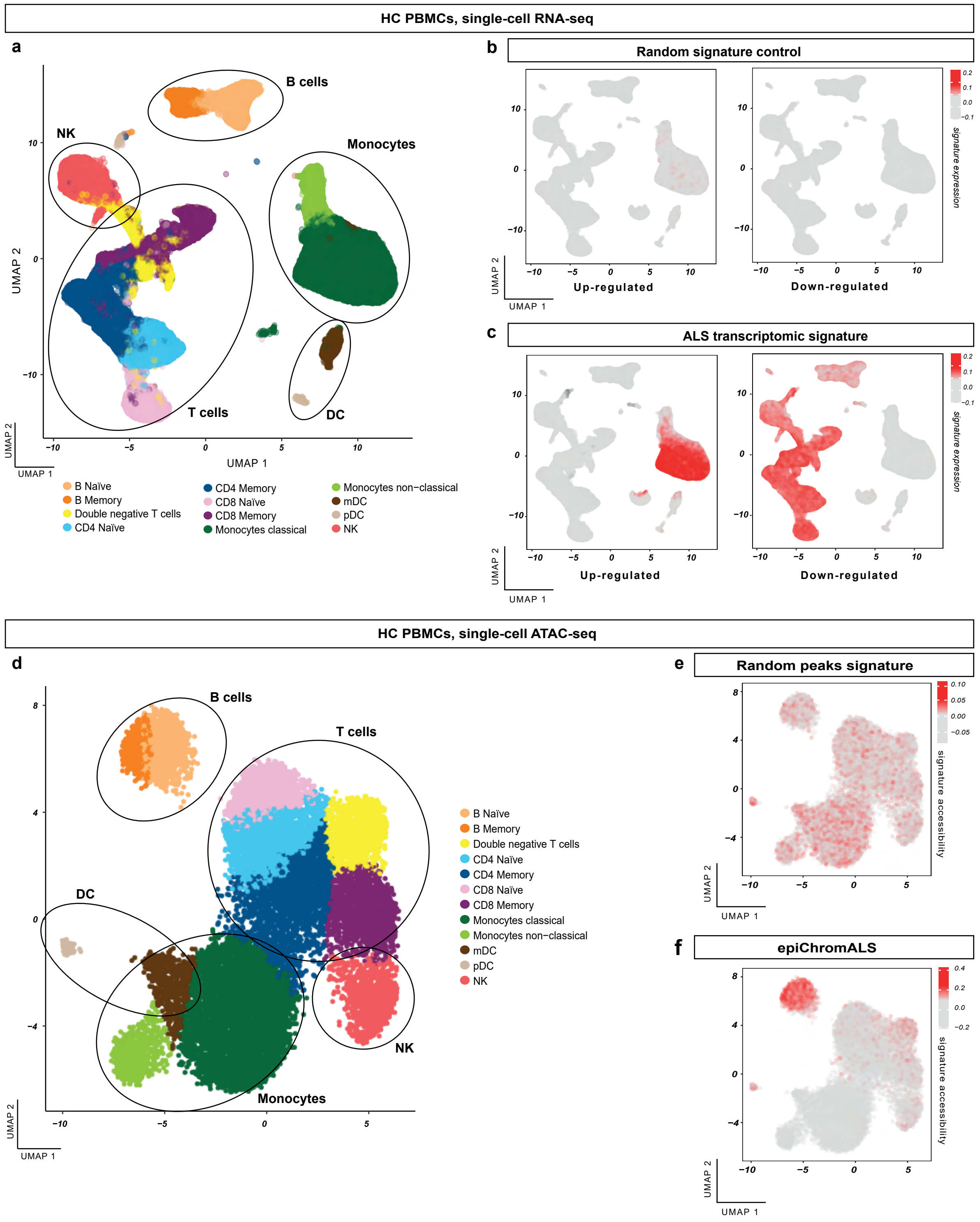

Supplementary Figure S12.

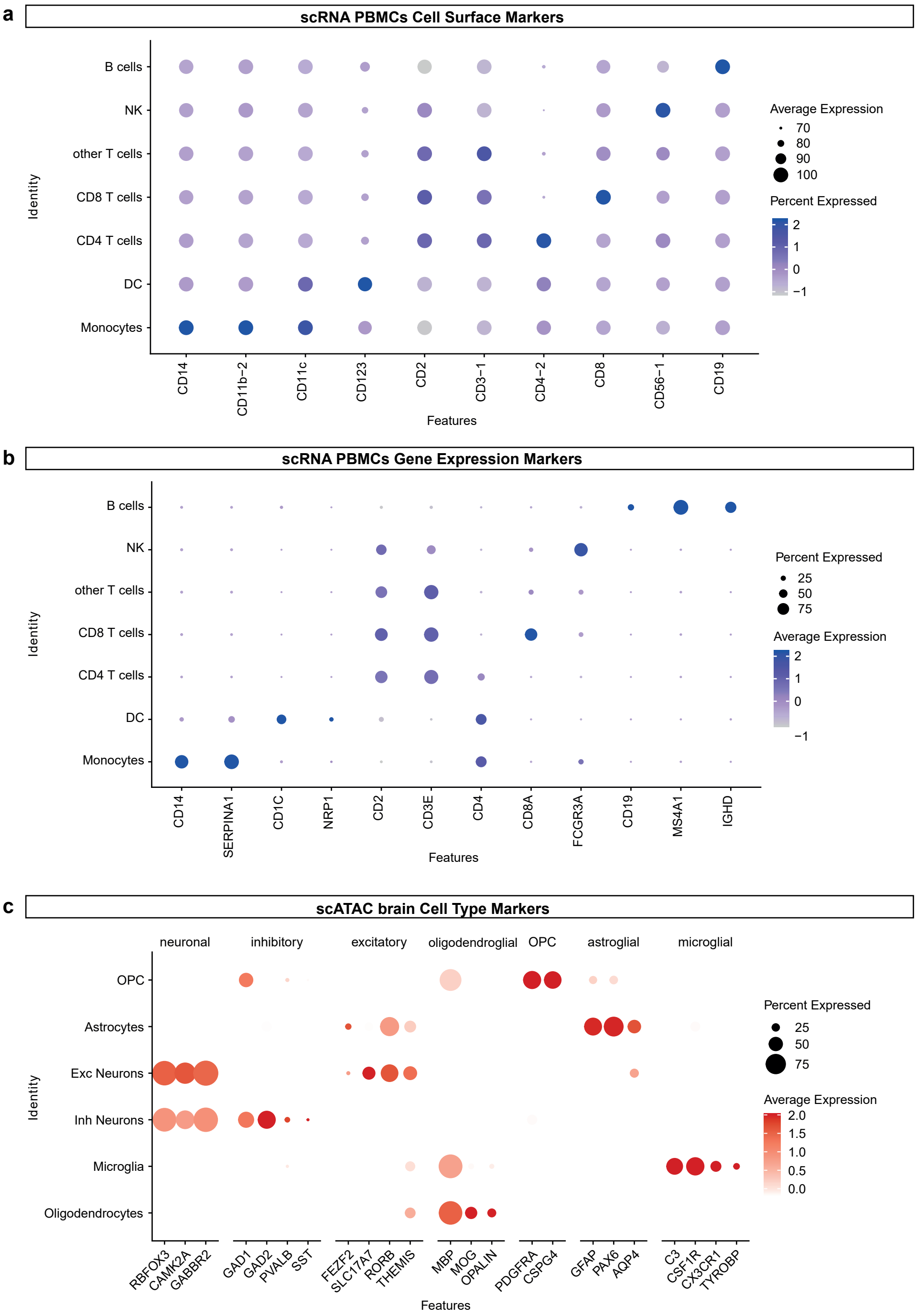

Supplementary Figure S13.

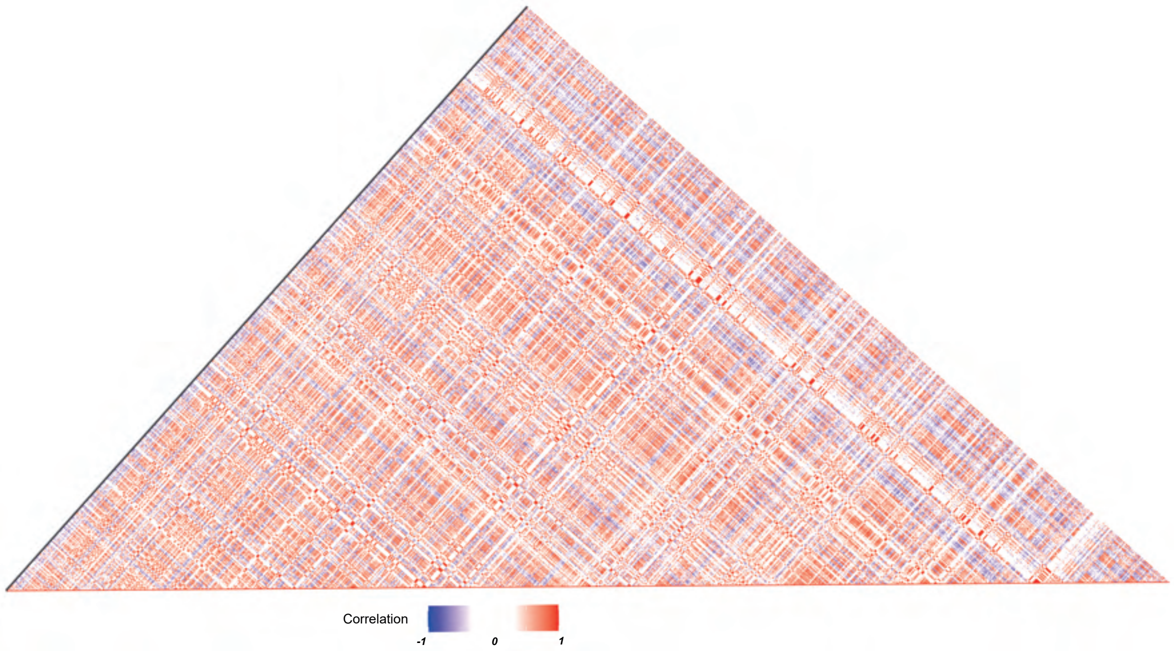

Supplementary Figure S14.

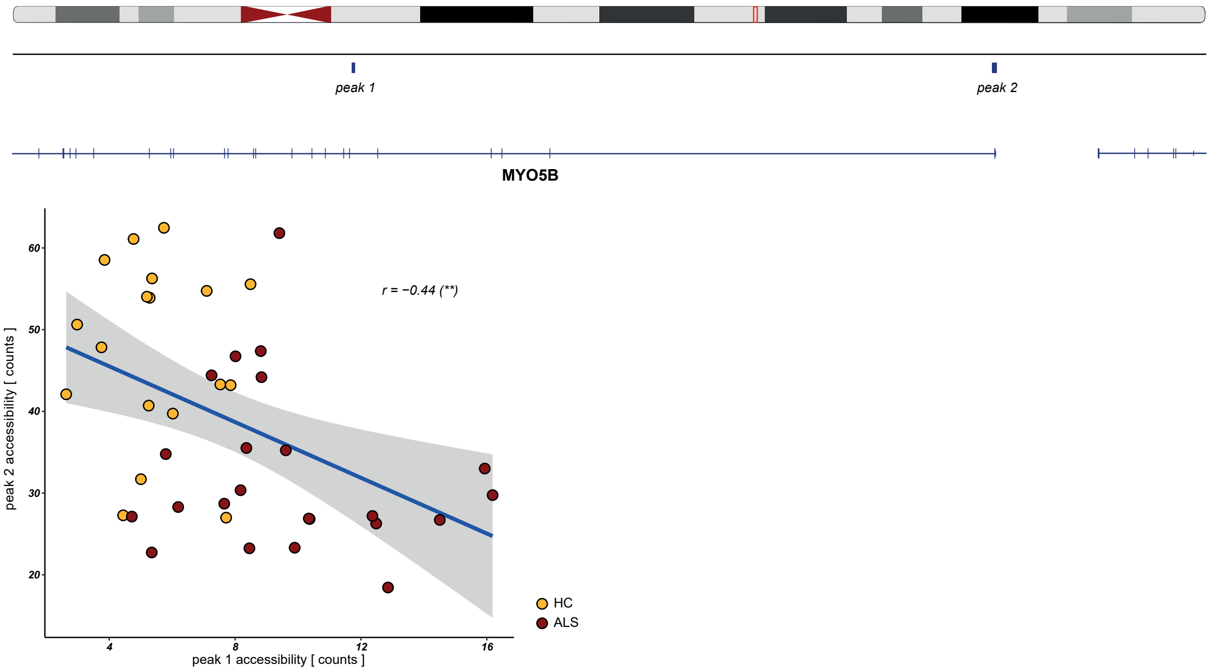

Supplementary Figure S15.

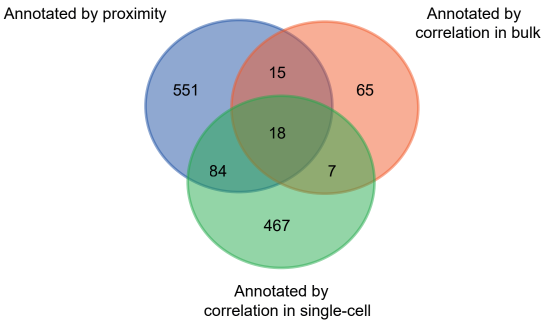

Supplementary Figure S16.

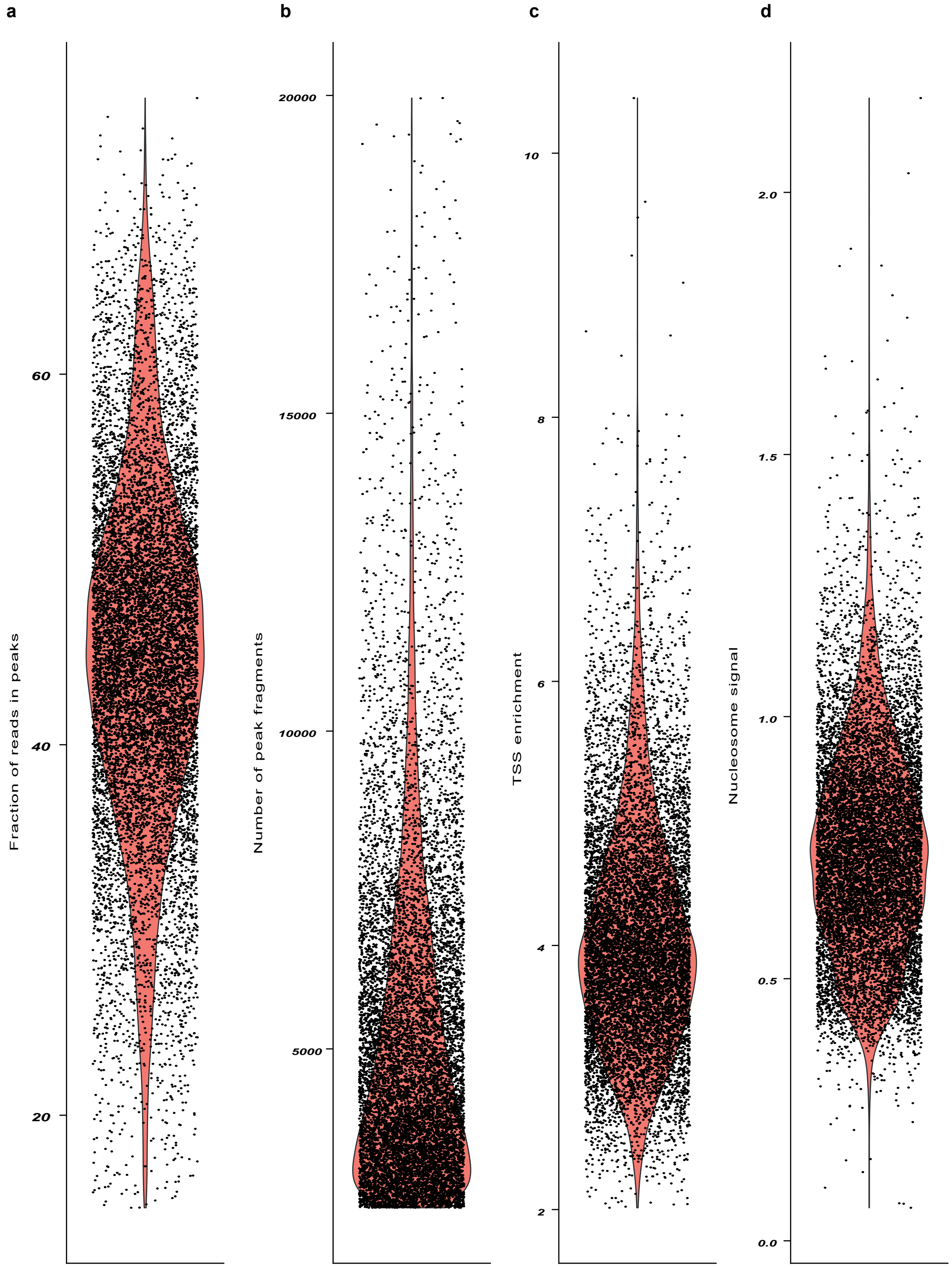

Supplement: Supplementary file 1 — Supplementary file1 (PDF 2702 KB) [file 18_2023_4769_MOESM1_ESM.pdf]
